# Mapping the metabolic reprogramming induced by sodium-glucose cotransporter 2 inhibition

**DOI:** 10.1172/jci.insight.164296

**Published:** 2023-04-10

**Authors:** Aviram Kogot-Levin, Yael Riahi, Ifat Abramovich, Ofri Mosenzon, Bella Agranovich, Liat Kadosh, Rachel Ben-Haroush Schyr, Doron Kleiman, Liad Hinden, Erol Cerasi, Danny Ben-Zvi, Ernesto Bernal-Mizrachi, Joseph Tam, Eyal Gottlieb, Gil Leibowitz

**Affiliations:** 1 Diabetes Unit and Endocrine Service, Hadassah-Hebrew University Medical Center, Jerusalem, Israel.; 2 The laboratory for Metabolism in Health and Disease, Ruth and Bruce Rappaport Faculty of Medicine, Technion-Israel Institute of Technology Haifa, Israel.; 3 Department of Developmental Biology and Cancer Research, Institute of Medical Research Israel-Canada, Faculty of Medicine, and; 4 Obesity and Metabolism Laboratory, Institute for Drug Research, School of Pharmacy, Faculty of Medicine, The Hebrew University of Jerusalem, Jerusalem, Israel.; 5 Department of Internal Medicine, Division of Endocrinology, Metabolism and Diabetes, Miller School of Medicine, University of Miami, Miami, Florida, USA.

**Keywords:** Metabolism, Therapeutics, Diabetes, Glucose metabolism, Signal transduction

## Abstract

Diabetes is associated with increased risk for kidney disease, heart failure, and mortality. Sodium-glucose cotransporter 2 inhibitors (SGLT2i) prevent these adverse outcomes; however, the mechanisms involved are not clear. We generated a roadmap of the metabolic alterations that occur in different organs in diabetes and in response to SGLT2i. In vivo metabolic labeling with ^13^C-glucose in normoglycemic and diabetic mice treated with or without dapagliflozin, followed by metabolomics and metabolic flux analyses, showed that, in diabetes, glycolysis and glucose oxidation are impaired in the kidney, liver, and heart. Treatment with dapagliflozin failed to rescue glycolysis. SGLT2 inhibition increased glucose oxidation in all organs; in the kidney, this was associated with modulation of the redox state. Diabetes was associated with altered methionine cycle metabolism, evident by decreased betaine and methionine levels, whereas treatment with SGLT2i increased hepatic betaine along with decreased homocysteine levels. mTORC1 activity was inhibited by SGLT2i along with stimulation of AMPK in both normoglycemic and diabetic animals, possibly explaining the protective effects against kidney, liver, and heart diseases. Collectively, our findings suggest that SGLT2i induces metabolic reprogramming orchestrated by AMPK-mTORC1 signaling with common and distinct effects in various tissues, with implications for diabetes and aging.

## Graphical abstract

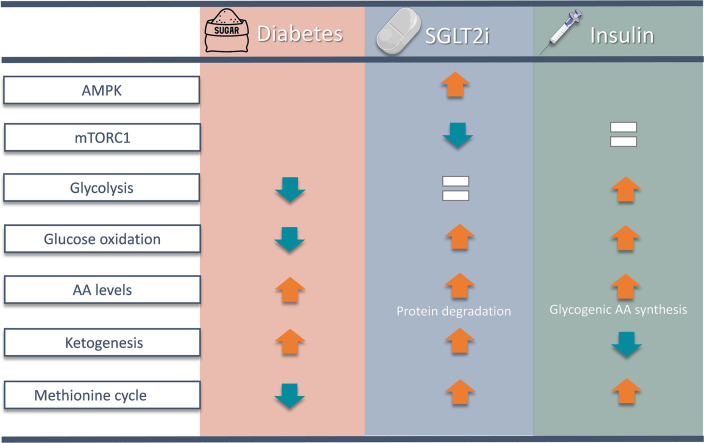

## Introduction

The kidney has an important role in regulating glucose homeostasis in mammals. Approximately 180 g/day glucose is filtered in the glomeruli, the vast majority being reabsorbed in kidney proximal tubule cells (KPTCs), mainly (90%) through sodium-glucose cotransporter 2 (SGLT2) (1–3). In diabetes, glucose reabsorption is increased, thereby aggravating the hyperglycemia (3). SGLT2 inhibitors (SGLT2i) induce glycosuria and are commonly used for the treatment of diabetes. Strikingly, large-scale trials consistently showed that SGLT2i effectively prevent the decline in kidney function and improve heart function in congestive heart failure in patients with and without diabetes; these improvements include the slowed progression to end-stage kidney disease, fewer hospitalizations for heart failure, and lower mortality (4–10). Early clinical studies suggest that SGLT2i are also beneficial in patients with nonalcoholic fatty liver disease (NAFLD) (11, 12). Interestingly, the SGLT2i canagliflozin has been shown to extend the lifespan in aged male rodents (13). These robust multiorgan beneficial effects of SGLT2i suggest that reduction of glucose load through increased glycosuria induces a systemic metabolic reprogramming that affects metabolism in distant organs.

Ferrannini and colleagues have shown that, in patients with type 2 diabetes, SGLT2i-induced glycosuria is associated with increased endogenous glucose production, enhanced insulin sensitivity, and shift of substrate utilization from carbohydrate to lipid (14, 15); it has been hypothesized that this metabolic shift mediates the beneficial cardiorenal effects of SGLT2i (2). According to this hypothesis, glycosuria reduces insulin secretion and glucose uptake for both oxidative and nonoxidative metabolism. This, in turn, results in a compensatory increase in lipolysis, which is partially mediated via FGF21, lipid oxidation, and ketogenesis as alternative fuels (2, 16). However, in nondiabetic subjects, the metabolic shift from glucose to lipid is small compared with patients with diabetes, whereas the cardiorenal beneficial effects are similar in both groups (14). Furthermore, in physiology, excess free fatty acid (FFA) delivery to insulin-sensitive tissues while fasting competes with glucose and causes insulin resistance, whereas treatment with SGLT2i is associated with improved insulin sensitivity (2, 15, 17). These paradoxical findings suggest that the metabolic reprogramming induced by SGLT2i is more complex than effects on fatty acid metabolism. Metabolomics on plasma and urine of patients with diabetes and rodent models of type 2 diabetes treated with SGLT2i showed alterations in metabolite profile (18–22); however, a comprehensive assessment of the metabolic response to SGLT2i in different organs is lacking.

Herein, we treated control mice and insulin-deficient hyperglycemic Akita mice with SGLT2i and used in vivo metabolic labeling with ^13^C-glucose followed by broad metabolomics and metabolic flux analyses simultaneously in the kidney, liver, heart, and plasma. We further performed targeted assessment of gene expression and activity of key metabolic enzymes and nutrient signaling pathways. We found that, in diabetes, both glycolysis and glucose oxidation are markedly impaired in the kidney, liver, and heart. SGLT2i decreased free glucose levels without improving glycolysis. Furthermore, in both normoglycemic and diabetic animals, treatment with SGLT2i resulted in accumulation of the glycolytic intermediates dihydroxyacetone phosphate (DHAP) and glyceraldehyde-3-phosphate (GA3P) in liver and kidney; this was associated with inhibition of the rate-limiting glycolytic enzyme pyruvate kinase in the liver. Importantly, SGLT2i increased glucose/pyruvate oxidation in the tricarboxylic acid (TCA) cycle, activated AMPK, and inhibited the nutrient sensor mTORC1 in all organs; this probably explains the protective effects on the kidney, heart, and liver (23–25).

## Results

### SGLT2i effects on glucose load, insulin secretion, and insulin sensitivity.

**Figure 1:**
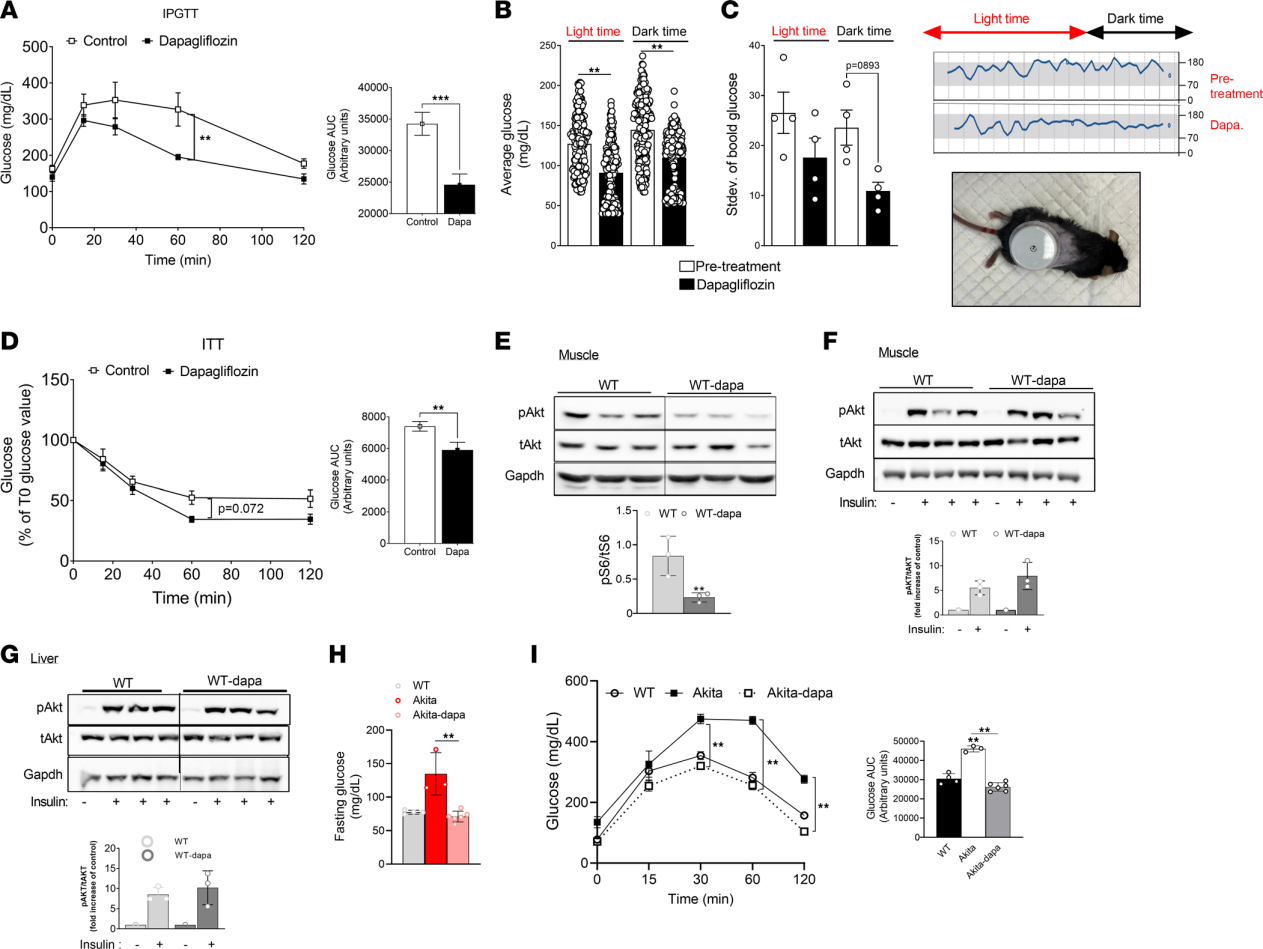
Metabolic effects of treatment with SGLT2i in WT and Akita mice. (**A**–**H**) Two-month-old WT mice fed on regular chow were treated with or without dapagliflozin (10 mg/kg/day in drinking water for 2 weeks) compared with untreated controls. (**A**) IPGTT; glucose (1.5 g/kg) was given i.p. after a 4-hour fast. (**B** and **C**) Continuous measurement of blood glucose using FreeStyle Libre flash monitoring system. Average glucose levels and glucose variability (Stdev) during the dark and light periods before and after adding dapagliflozin and a representative plot are shown. (**D**) Insulin tolerance test; insulin (1 U/kg) was injected to mice after a 4-hour fast with consecutive measurements of blood glucose. Results are expressed as percentage of glucose levels at time 0. Blood glucose levels at baseline were 137.7 ± 18.4 and 127.1 ± 23.3 mg/dL in control and dapagliflozin treated mice, respectively. (**E**) Steady-state mTORC1 activity in muscle, evident by S6 phosphorylation; lanes were run on the same gel but were noncontiguous. (**F** and **G**) Insulin-stimulated AKT phosphorylation is shown in muscle and liver. Insulin (3 U/kg mice) was injected i.p. after an overnight fast. Gastrocnemius muscle and the liver were isolated and extracted after 5 minutes, and AKT phosphorylation was analyzed by Western blotting. Data represent the mean ± SEM of 3–8 mice per group. (**H** and **I**) Two-month-old Akita mice were fed on regular chow and treated without or with dapagliflozin (10 mg/kg/day in drinking water) for 1 week. (**H**) Overnight fasting blood glucose levels. (**I**) IPGTT was performed after an overnight fast. Data represent the mean ± SEM of 3–8 mice per group. Data were analyzed by unpaired 2-tailed Student’s *t* test (**A**–**E**) or by 2-way ANOVA (**F**–**I**). *P < 0.05, **P < 0.01, ***P < 0.001.

We have previously shown in Akita mice, a type 1 diabetes model, that treatment with the SGLT2i dapagliflozin prevented the development of diabetic kidney disease, thus mimicking the SGLT2i effect in patients with diabetes (23). We tested the effects of dapagliflozin (10 mg/kg in drinking water) on blood glucose in WT mice that were fed for 2 weeks on regular chow. Fasting and fed blood glucose, insulin, and glucagon were similar in all groups (Supplemental Figure 1; supplemental material available online with this article; https://doi.org/10.1172/jci.insight.164296DS1). I.p. glucose tolerance test (IPGTT) showed that glucose excursions were reduced in mice treated with dapagliflozin (Figure 1A). We also tested the SGLT2i effect on blood glucose response to stimulation of hepatic glucose production. Treatment with dapagliflozin abrogated the glucose response to glucagon, pyruvate, and glycerol (Supplemental Figure 1). We further performed continuous blood glucose monitoring using the FreeStyle Libre flash monitoring system, which was inserted into the back of WT mice under anesthesia, and we monitored blood glucose during the dark and light periods. Baseline glucose levels were recorded for 2 days after recovery from surgery and then for 3 days while dapagliflozin was administered. Treatment with dapagliflozin decreased average blood glucose during the day and night, and it decreased glucose variability while eating during the dark period (Figure 1, B and C), indicating reduction of the glucose load even in the physiological range. Insulin tolerance tests (ITT) showed that insulin sensitivity of mice treated with dapagliflozin was improved (Figure 1D). The muscle and the liver are responsible for the majority of whole-body insulin-dependent glucose disposal. SGLT2i may affect insulin sensitivity by inhibiting mTORC1, which negatively regulates insulin signaling via IRS1/PI3K/AKT. We therefore tested mTORC1 activity in striated muscle and the effects of i.p. insulin injection on AKT phosphorylation. Treatment with dapagliflozin reduced steady-state mTORC1 activity in the muscle (Figure 1E); however, insulin-stimulated AKT phosphorylation after an overnight fast was similar in the muscle and liver (Figure 1, F and G), suggesting that the greater hypoglycemic effect of insulin in mice treated with SGLT2i resulted from excess glucose loss in the urine, rather than enhanced insulin sensitivity in target organs. Finally, we tested the SGLT2i effects on blood glucose in diabetic Akita mice by IPGTT. Dapagliflozin decreased fasting blood glucose and markedly reduced the glucose excursion following i.p. glucose injection; glucose tolerance in Akita mice treated with dapagliflozin was similar to that of WT mice (Figure 1, H and I). In summary, treatment with SGLT2i reduced glucose load in both normoglycemic and diabetic mice, and it improved glucose tolerance and decreased the activity of the central nutrient sensor mTORC1 in muscle, suggesting systemic metabolic reprogramming.

### Effects of diabetes and treatment with SGLT2i on metabolomics and glucose fluxes in the kidney cortex, liver, and heart.

We treated WT mice and Akita mice with dapagliflozin for 1 week, followed by metabolic labeling with ^13^C-glucose, and we performed metabolomics and metabolic flux analyses in kidney cortex extracts, liver, heart, and plasma. Labeled glucose was given in the fed state, after food deprivation for 1 hour; as an additional control, overnight-fasted WT animals were used. In addition, we tested the effects of treatment with insulin on nutrient metabolism and signaling in Akita mice. The full list of metabolites in the different tissues, and in the plasma of diabetic animals and/or in response to treatment with dapagliflozin, is shown in Supplemental Tables 1–4. It has been previously suggested that chronic treatment with SGLT2i induce a metabolic shift of substrate utilization from carbohydrate to lipid, hence mimicking fasting. Principal component analysis (PCA) showed that metabolite clustering in the liver, kidney, and plasma, but not in the heart of overnight-fasting WT animals, was distinct from that of WT and diabetic animals with and without dapagliflozin treatment (Supplemental Figure 2, A–D). Diabetes and treatment with dapagliflozin altered metabolite distribution with partial overlap between groups. A heatmap showing the top 50 metabolites that were differentially present in the various groups is presented in Supplemental Figure 3, A–D. In the diabetic kidney, carbohydrates and glucose amines, including glucose, fructose-6 phosphate (F6P), trehalose, sorbitol/mannitol, galactosamine, and glucosamine were among the top upregulated metabolites, and treatment with dapagliflozin decreased their levels (Supplemental Figure 3, A–D). This finding is consistent with a previous report suggesting that the diabetic kidney is ineffective in discarding glucose metabolites that promote the development of diabetic kidney disease (26). Fatty acids, including stearidonic acid, palmitoleic acid, myristic acid, and myristoleic acid, were increased in the liver and kidney of fasted mice but not in the fed state in untreated and dapagliflozin-treated WT and Akita mice.

Interestingly, treatment with dapagliflozin increased the level of the glycolytic intermediates DHAP and GA3P in both WT and diabetic kidneys and livers (Supplemental Figure 3, A–D). In the kidney, these were the only metabolites in the top 50 heatmap that were upregulated by dapagliflozin in both normoglycemic and diabetic animals, whereas in the liver, there were several metabolites of the one carbon metabolism pathway, including serine metabolites and betaine, that were increased in both groups. Pathway enrichment analysis shows that glycolysis and one carbon metabolism pathways, including glycine, serine, betaine, and methionine metabolism, were among the pathways that were enriched the most in diabetic versus WT animals (Supplemental Figure 4). Treatment of WT and Akita mice with dapagliflozin was associated with enrichment of these pathways, as well as enrichment of nucleic acids, amino acids, and urea cycle metabolism, and it was associated with the Warburg effect, which is typically associated with cancer (Supplemental Figures 4 and 5). In addition, treatment with dapagliflozin was associated with enrichment of homocysteine degradation in the diabetic kidney and heart.

### Dysregulated glucose metabolism in the diabetic kidney and effects of treatment with SGLT2i.

**Figure 2:**
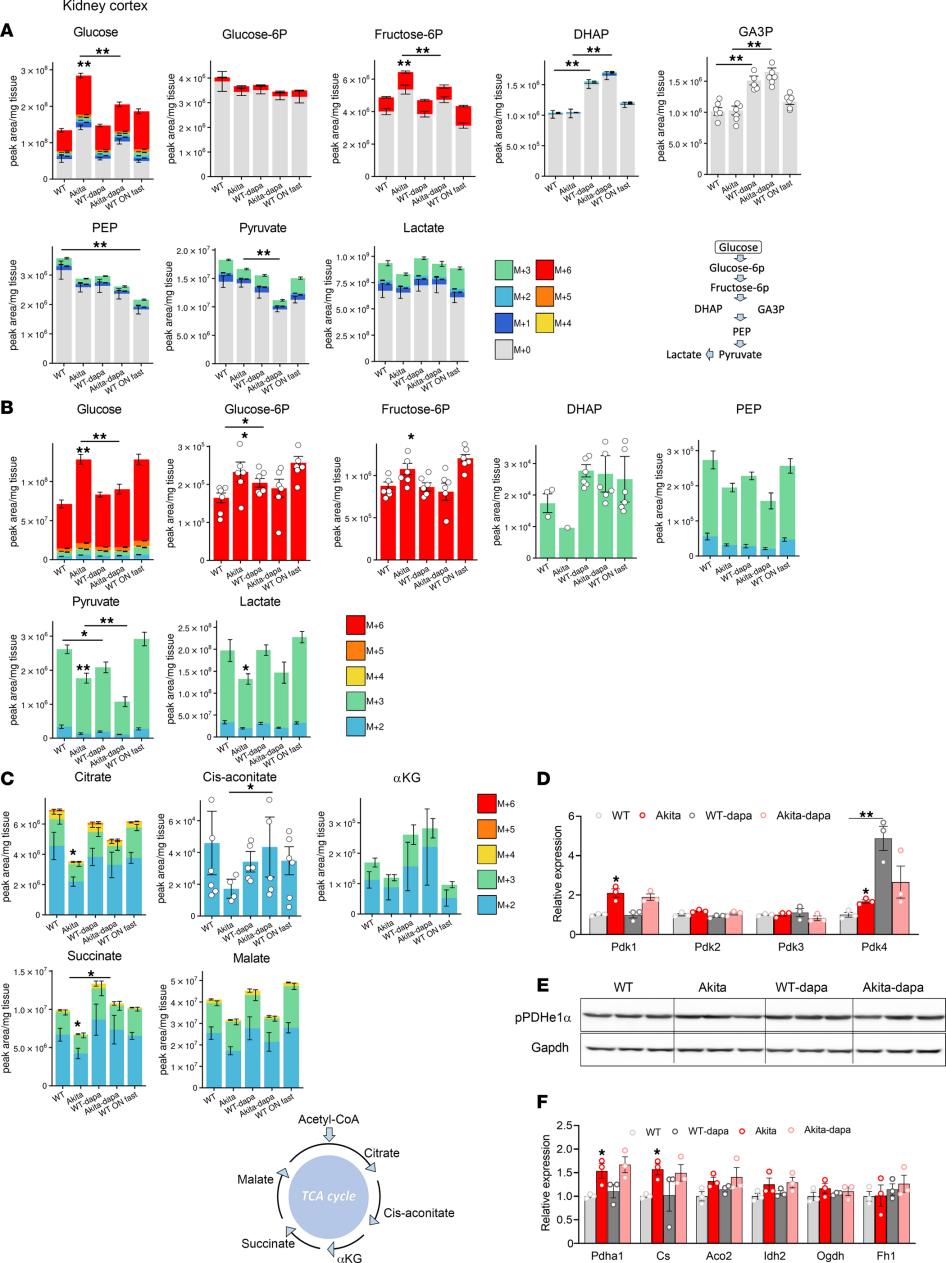
Effect of diabetes and of SGLT2i on glycolysis and glucose oxidation in kidney cortex extracts of WT and Akita mice. WT and Akita mice were treated with and without dapagliflozin for 1 week, followed by ^13^C-glucose injections and metabolomics and metabolic flux analyses. (**A**) Unlabeled levels of glycolytic intermediates in kidney cortex extracts. Shown are the relative levels of unlabeled (^12^C) glucose and ^13^C-labeled glycolytic intermediates. A schematic representation of glycolysis is shown. (**B**) Relative abundance of ^13^C-labeled glucose and glycolytic intermediates in kidney cortex. (**C**) Relative abundance of ^13^C-labeled tricarboxylic acid (TCA) cycle metabolites in kidney cortex. A schematic representation of the TCA cycle is shown. (**D**) mRNA levels of Pdk1-4 in kidney cortex. (**E**) Western blotting on kidney extracts for phosphorylated pyruvate dehydrogenase α1 (pPDHe1α) and GAPDH. (**F**) mRNA expression of TCA cycle enzymes in kidney cortex. Data represent the mean ± SEM, *n* = 3–6 mice per group. For statistical analysis, we used the sum of all ^13^C isotopologues for each metabolite or the unlabeled + ^13^C-labeled metabolites. Data were analyzed by 2-way ANOVA. *P < 0.05, **P < 0.01.

We analyzed unlabeled and ^13^C-labeled metabolites in the kidney cortex. Both unlabeled and labeled glucose were higher in diabetic animals; treatment with dapagliflozin attenuated free glucose accumulation (Figure 2, A and B); these alterations may reflect glucose levels in the plasma left in the kidney and, potentially, cellular glucose uptake. Intracellularly, glucose is rapidly phosphorylated by hexokinases. The levels of proximal glycolytic intermediates (G6P and F6P) were increased in diabetic kidneys; this was attenuated by dapagliflozin. Distal glucose–derived glycolytic intermediates (^13^C-labeled pyruvate and lactate) were decreased in the diabetic kidney (Figure 2B). Treatment with SGLT2i increased DHAP and GA3P levels (Figure 2A) and further decreased ^13^C-labeled pyruvate without affecting lactate levels. The expression of *Enolase 1*, but not of other glycolytic enzymes, was increased in diabetes, whereas treatment of diabetic animals with dapagliflozin further increased the expression of *Enolase 1*, *Aldolase B*, and *Pkm2* (Supplemental Figure 6A). Of note, despite the upregulation of *Pkm2* expression in response to treatment with dapagliflozin, pyruvate kinase activity was not affected by SGLT2i (Supplemental Figure 6B). Collectively, the alterations in glucose-derived pyruvate and lactate suggest that glycolysis is dysregulated in diabetes and that treatment with SGLT2i failed to correct glycolysis in the diabetic kidney.

Next, we analyzed glucose oxidation in the diabetic kidney and the response to SGLT2i. In diabetic animals, glucose-derived TCA cycle intermediates — including citrate and succinate (*P* < 0.05), *cis*-aconitate, α-ketoglutarate, and malate (not significant) — were decreased, whereas treatment with dapagliflozin increased the levels of ^13^C-labeled TCA cycle intermediates (citrate, *cis*-aconitate, α-ketoglutarate, and succinate) (Figure 2C), indicating greater supply of glucose-derived metabolites for oxidation in the TCA cycle. Pyruvate entry to the TCA cycle is regulated by pyruvate dehydrogenase kinases (PDKs). The expression of *Pdk1* and *Pdk4* was increased in diabetic kidneys and was not affected by dapagliflozin (Figure 2D), suggesting that impaired glucose oxidation might be partially mediated via increased PDK gene expression and that treatment with SGLT2i increased glucose oxidation, irrespective of PDKs gene expression. PDHe1α phosphorylation at Ser 293 was not increased in Akita mice and was not affected by treatment with dapagliflozin (Figure 2E), suggesting that pyruvate entry to the TCA cycle is regulated by phosphorylation at a different site, by allosteric modification of pyruvate dehydrogenase (PDH), and/or by alterations in its intracellular localization.

Unlabeled TCA cycle intermediates and ATP levels were similar in diabetic and WT kidneys and were not affected by dapagliflozin (except for increased succinate in diabetic animals treated with dapagliflozin) (Supplemental Figure 6C); therefore, alternative fuels, such as fatty acids and amino acids (e.g., glutamate), can compensate for impaired glucose oxidation in diabetes. The expression of TCA enzymes (*PDH1a* and *Citrate synthase*) was increased in diabetic kidneys and was not affected by treatment with dapagliflozin, further suggesting that, during early stages of diabetes, impaired glucose oxidation is not mediated via downregulation of TCA cycle enzyme gene expression (Figure 2F).

Enhanced glucose oxidation can increase NADH and promote cytosolic NADPH generation via the isocitrate–α-ketoglutarate shuttle and potentially other anaplerosis/cataplerosis shuttles. Consistently, NADH/NAD^+^ and NADPH/NADP^+^ ratios were increased in the kidney of diabetic animals that were treated with dapagliflozin compared with Akita controls (Supplemental Figure 6, D and E). The intracellular lactate/pyruvate concentration ratio is proportional to free cytosolic NADH/NAD^+^ and is a common biomarker of redox; the lactate/pyruvate ratio was also increased by dapagliflozin (Supplemental Figure 6F).

Collectively, these findings demonstrate that treatment with SGLT2i improves glucose oxidation in the TCA cycle, despite the limited availability of pyruvate. This can increase the reduction potential, which is beneficial under conditions of diabetes-induced oxidative stress.

### Dysregulated glucose metabolism in the diabetic liver and effects of treatment with SGLT2i.

**Figure 3:**
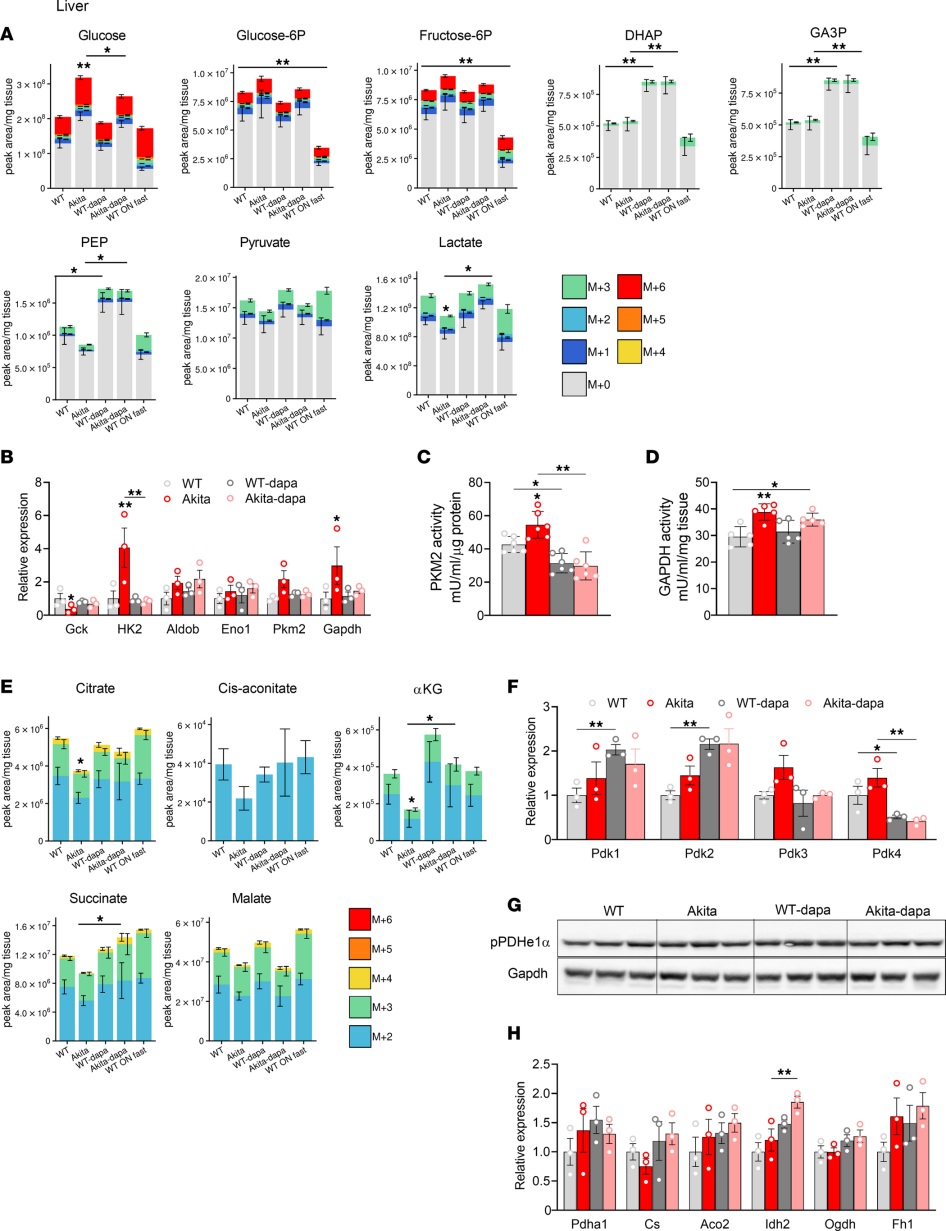
Effect of diabetes and of SGLT2i on glycolysis and glucose oxidation in liver of WT and Akita mice. WT and Akita mice were treated with and without dapagliflozin for 1 week, followed by ^13^C-glucose injections and metabolomics and metabolic flux analyses. (**A**) Unlabeled levels of glycolytic intermediates in liver extracts. Shown are the relative levels of unlabeled (^12^C) glucose and ^13^C-labeled glycolytic intermediates. (**B**) mRNA expression of glycolytic enzymes in liver. (**C**) Pyruvate kinase activity in liver homogenate. (**D**) GAPDH activity in liver homogenate. (**E**) Relative abundance of ^13^C-labeled tricarboxylic acid (TCA) cycle metabolites in liver. (**F**) mRNA levels of Pdk1–4 in liver. (**G**) Western blotting on liver extracts for phosphorylated pyruvate dehydrogenase α1 (pPDHe1α) and GAPDH. (**H**) mRNA expression of TCA cycle enzymes in liver. Data represent the mean ± SEM, *n* = 3–6 mice per group. For statistical analysis, we used the sum of all ^13^C isotopologues for each metabolite or the unlabeled + ^13^C-labeled metabolites. Data were analyzed by 2-way ANOVA. *P < 0.05, **P < 0.01.

In diabetic animals, the levels of glycolytic intermediates remained unchanged in the liver, except for a modest decline of the distal glycolytic intermediates phosphoenolpyruvate (PEP), pyruvate, and lactate (*P* < 0.05) (Figure 3A and Supplemental Figure 7A). Glucokinase is a high Km enzyme that regulates glycolysis in the liver. In diabetes, the *Glucokinase* gene expression was decreased, whereas *Hexokinase 2*, *Gapdh*, and *Pkm2* (not significant) were increased; these alterations were reversed by dapagliflozin (Figure 3B). Treatment with dapagliflozin markedly increased DHAP, GA3P, and PEP in both WT and diabetic animals (Figure 3A).

Pyruvate kinase and GAPDH are rate-limiting enzymes in glycolysis that have been implicated in the Warburg effect and cancer (27). Both pyruvate kinase and GAPDH activities were increased in the diabetic liver (Figure 3, C and D), and pyruvate kinase activity decreased in response to treatment with dapagliflozin in both normoglycemic and diabetic animals (Figure 3C), consistent with the upregulation of glycolytic intermediates that reside upstream to pyruvate kinase.

Like in the kidney, the levels of glucose-derived TCA cycle intermediates (citrate, *cis*-aconitate, α-ketoglutarate, and succinate) were decreased in diabetes, indicating a reduced supply of glucose metabolites for oxidation in the TCA cycle; these alterations were reversed by treatment with SGLT2i (Figure 3E and Supplemental Figure 7B). Interestingly, *Pdk4* gene expression was increased in diabetes and decreased by dapagliflozin in both WT and diabetic animals (Figure 3F). Like in the kidney, hepatic PDHe1α phosphorylation remained unchanged in diabetes and in response to SGLT2i (Figure 3G). In addition, the expression of *Isocitrate dehydrogenase 2* (*Idh2*), but not of other TCA cycle genes, was increased by treatment with dapagliflozin (Figure 3H). Enhanced glucose oxidation in response to treatment with dapagliflozin was associated with increased ATP in WT animals and also in diabetic animals (though not significantly) (Supplemental Figure 7B).

Overall, these findings suggest that glycolysis is dysregulated in diabetes and that SGLT2i inhibit hepatic glycolysis through inhibition of pyruvate kinase. Furthermore, treatment with SGLT2i may enhance glucose oxidation despite the limited supply of pyruvate through inhibition of *Pdk4* gene expression and upregulation of *Idh2*.

### Dysregulated glucose metabolism in the diabetic heart and effects of treatment with SGLT2i.

**Figure 4:**
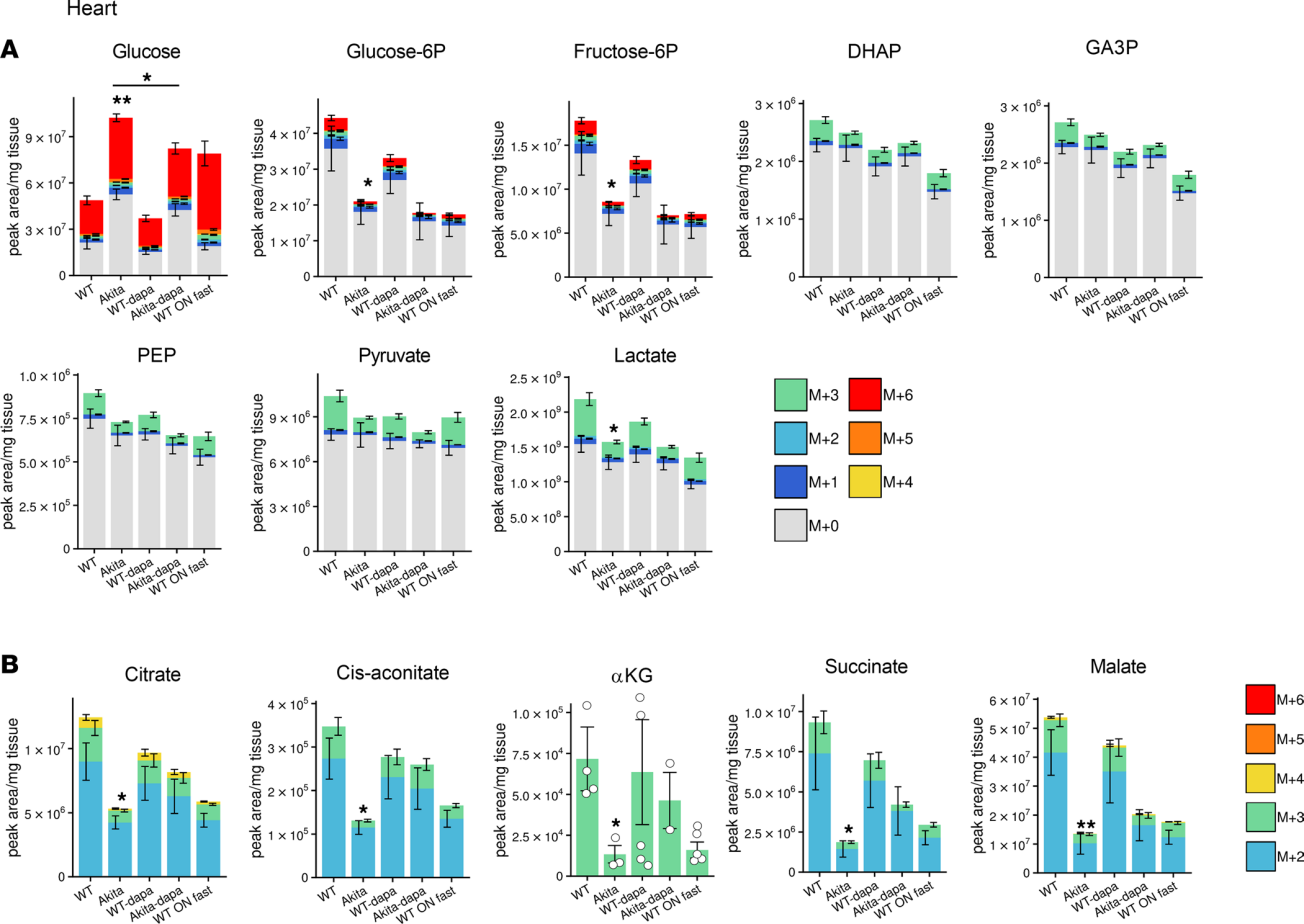
Effect of diabetes and of SGLT2i on glycolysis and glucose oxidation in heart of WT and Akita mice. WT and Akita mice were treated with and without dapagliflozin for 1 week, followed by ^13^C-glucose injections and metabolomics and metabolic flux analyses. (**A**) Unlabeled levels of glycolytic intermediates in heart extracts. Shown are the relative levels of unlabeled (^12^C) glucose and ^13^C-labeled glycolytic intermediates. (**B**) Relative abundance of ^13^C-labeled tricarboxylic acid (TCA) cycle metabolites in heart. Data represent the mean ± SEM, *n* = 6 mice per group. For statistical analysis, we used the sum of all ^13^C isotopologues for each metabolite or the unlabeled + ^13^C-labeled metabolites. Data were analyzed by 2-way ANOVA. *P < 0.05, **P < 0.01.

Free glucose was increased in the diabetic heart; however, all glucose-derived glycolytic intermediates were markedly reduced in the diabetic heart and were further decreased by dapagliflozin, showing that, in the diabetic heart, glycolysis is impaired and is not rescued by SGLT2i (Figure 4A and Supplemental Figure 8A). In diabetic hearts, glucose-derived TCA cycle intermediates, including citrate, *cis*-aconitate, α-ketoglutarate, succinate, and malate, were all decreased (Figure 4B), indicating that both glucose utilization and oxidation are markedly impaired. Intriguingly, neither the expression of glycolytic, TCA cycle enzymes and PDKs, nor PDHe1α phosphorylation were altered in diabetes (Supplemental Figure 8, B–E).

Unlabeled TCA cycle intermediates, including acetyl-CoA, citrate, *cis*-aconitate, and α-ketoglutarate levels, were increased along with preserved ATP, suggesting a shift from glucose oxidation to fatty acid and/or ketone body oxidation that can compensate for impaired glucose oxidation (Supplemental Figure 8F). Like in the diabetic kidney and liver, treatment of diabetic animals with dapagliflozin enhanced glucose oxidation, supported by the finding that the levels of all glucose-derived TCA cycle intermediates were similar in dapagliflozin-treated Akita mice and in WT animals (Figure 4B).

### Effects of insulin therapy on glucose metabolism in diabetic organs.

**Figure 5:**
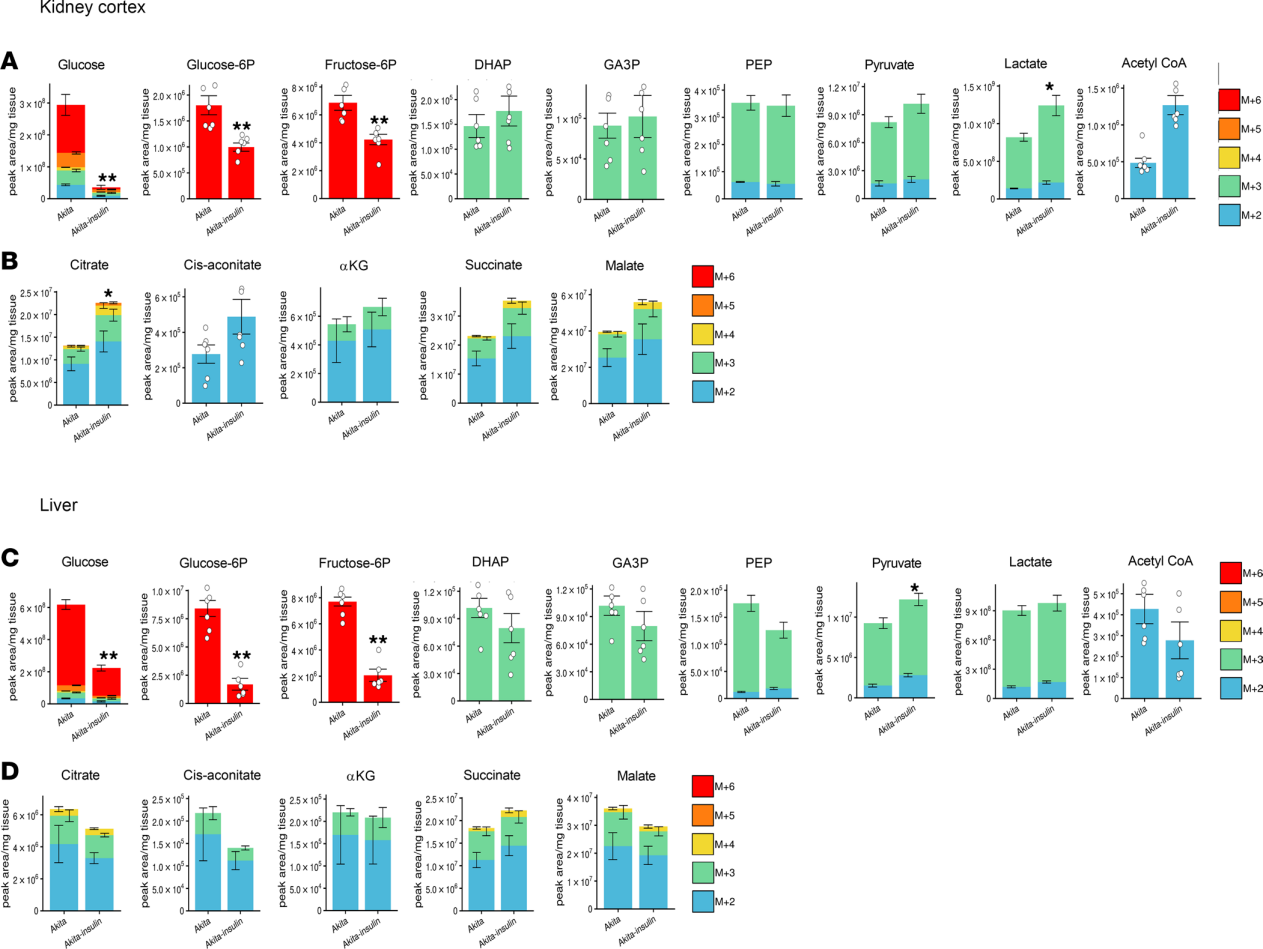
Effect of insulin on glycolysis and glucose oxidation in kidney cortex and liver extracts of Akita mice. Akita mice were treated with and without insulin for 5 days followed by ^13^C-glucose injections and metabolomics and metabolic flux analyses. (**A** and **B**) ^13^C-labeled glycolytic intermediates tricarboxylic acid (TCA) cycle metabolites in kidney cortex. (**C** and **D**) ^13^C-labeled glycolytic intermediates and TCA cycle intermediates in liver. Data represent the mean ± SEM, *n* = 6 mice per group. For statistical analysis, we used the sum of all ^13^C isotopologues for each metabolite. Data were analyzed by unpaired 2-tailed Student’s *t* test. *P < 0.05, **P < 0.01.

**Figure 6:**
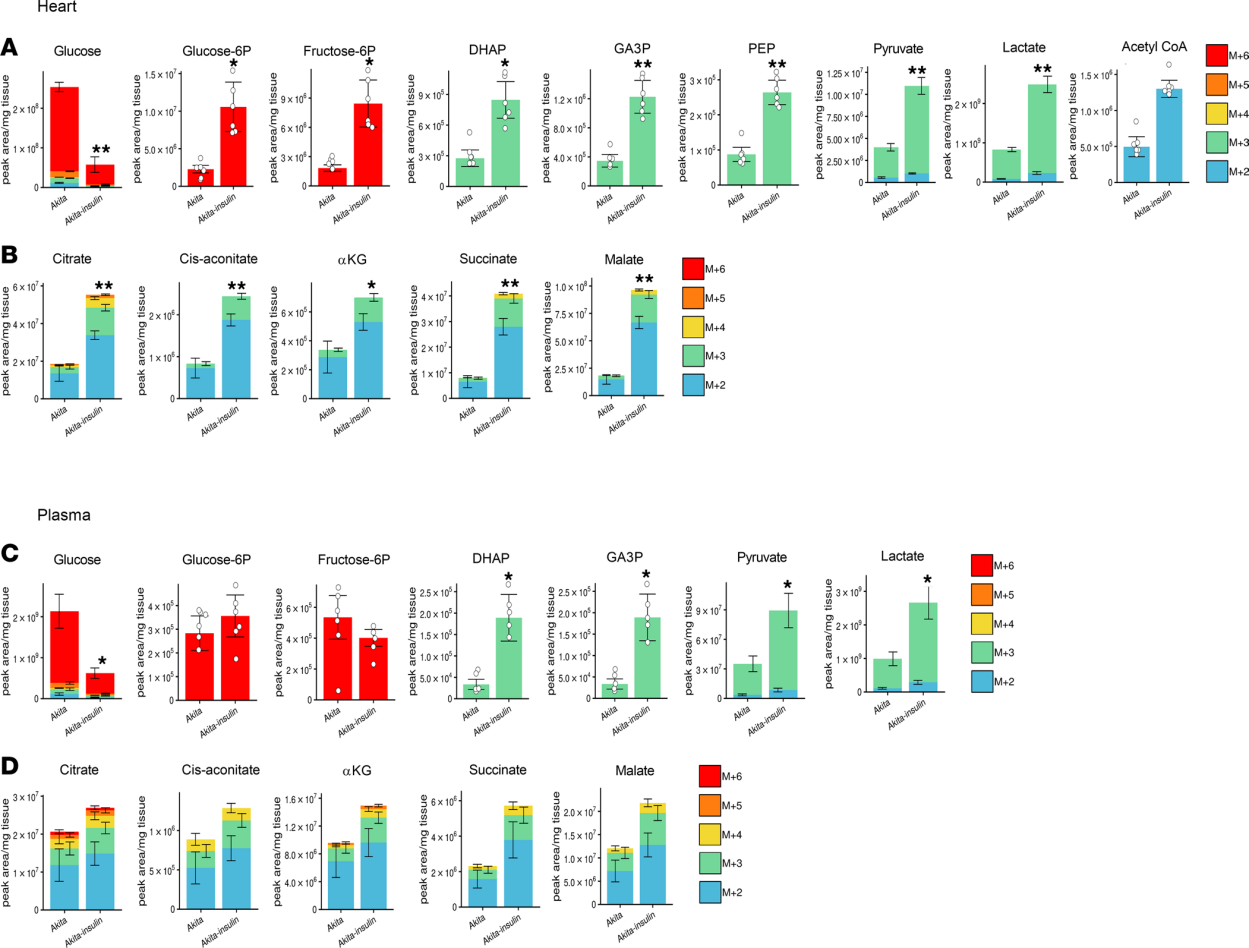
Effect of insulin on glycolysis and glucose oxidation in heart and plasma extracts of Akita mice. Akita mice were treated with and without insulin for 5 days followed by ^13^C-glucose injections and metabolomics and metabolic flux analyses. (**A** and **B**) ^13^C-labeled glycolytic and TCA cycle metabolites in the heart. (**C** and **D**) ^13^C-labeled glycolytic and TCA cycle intermediates in plasma. Data represent the mean ± SEM, *n* = 6 mice per group. For statistical analysis, we used the sum of all ^13^C isotopologues for each metabolite. Data were analyzed by unpaired 2-tailed Student’s *t* test. *P < 0.05, **P < 0.01.

Part of the metabolic effects of SGLT2i can result from reduction of hyperglycemia/glucotoxicity. We therefore tested the effects of insulin therapy on metabolomics and metabolic fluxes in diabetic Akita mice. We followed the same protocol that was used for testing the effects of SGLT2i. As expected, insulin effectively reduced glycemia and tissue glucose levels in the kidney, liver, and heart (Figure 5 and Figure 6). In contrast to SGLT2i, insulin markedly stimulated glycolysis, as shown by increased pyruvate and/or lactate levels; this was accompanied by increased glucose-derived TCA cycle metabolites in the kidney and heart but not in the liver (Figure 5 and Figure 6), suggesting increased glucose oxidation. Increased supply of glucose-derived TCA cycle fuels was most prominent in the heart.

Collectively, our findings show that lowering glycemia by either SGLT2i or insulin increases glucose oxidation in the kidney and heart; however, SGLT2i or insulin have contradictory effects on glycolysis, indicating dissimilar effects on glucose metabolism.

### SGLT2i effects on fatty acid and ketone body metabolism.

Prolonged fasting induces lipolysis and ketogenesis. As expected, FFA and β-hydroxybutyrate (βHB) levels were increased in the plasma as well as in the liver and kidney of fasted mice (Supplemental Figure 9, A–C). SGLT2i can enhance lipolysis and promote ketogenesis under conditions of prolonged starvation and/or insulin deficiency. The current analysis on dapagliflozin’s effect was performed in fed mice (1 hour of food deprivation). Consistently, there were only minor alterations in fatty acids and ketone bodies in WT mice treated with dapagliflozin. In Akita mice, βHB was increased, and the levels were like those obtained in WT mice under fasting. We assume that the persistent secretion of a small amount of insulin in young Akita mice was sufficient to restrain lipolysis and ketogenesis, hence preventing full-blown ketoacidosis, which is rare in this model. Diabetes was associated with a 6-fold increase in the expression of the fatty acid transporter CD36 in the liver (Supplemental Figure 9D) but not in the kidney or heart (not shown), suggesting that increased fatty acid uptake by the liver induces energy production under conditions of restrained glucose oxidation. Dapagliflozin, which improved glucose oxidation, prevented the upregulation of CD36 and did not further increase βHB (Supplemental Figure 9, D and E). The majority of ketone bodies are derived from fatty acid oxidation. Intriguingly, we found that treatment with dapagliflozin consistently increased the fraction of ^13^C-labeled βHB in both normoglycemic and in diabetic Akita mice, suggesting that increased glucose oxidation induced by SGLT2i is associated with a shift of glucose/pyruvate-derived acetyl CoA for ketogenesis (Supplemental Figure 9E). As expected, insulin markedly decreased βHB, and reduced the ^13^C-labeled fraction, indicating inhibition of ketogenesis (Supplemental Figure 9F). It has been previously suggested that SGLT2i increase lipolysis via FGF21 (2, 16); FGF21 was decreased in Akita mice compared with controls. However, treatment with dapagliflozin did not affect FGF21 levels (Supplemental Figure 10).

### SGLT2i effects on hepatic one carbon metabolism.

**Figure 7:**
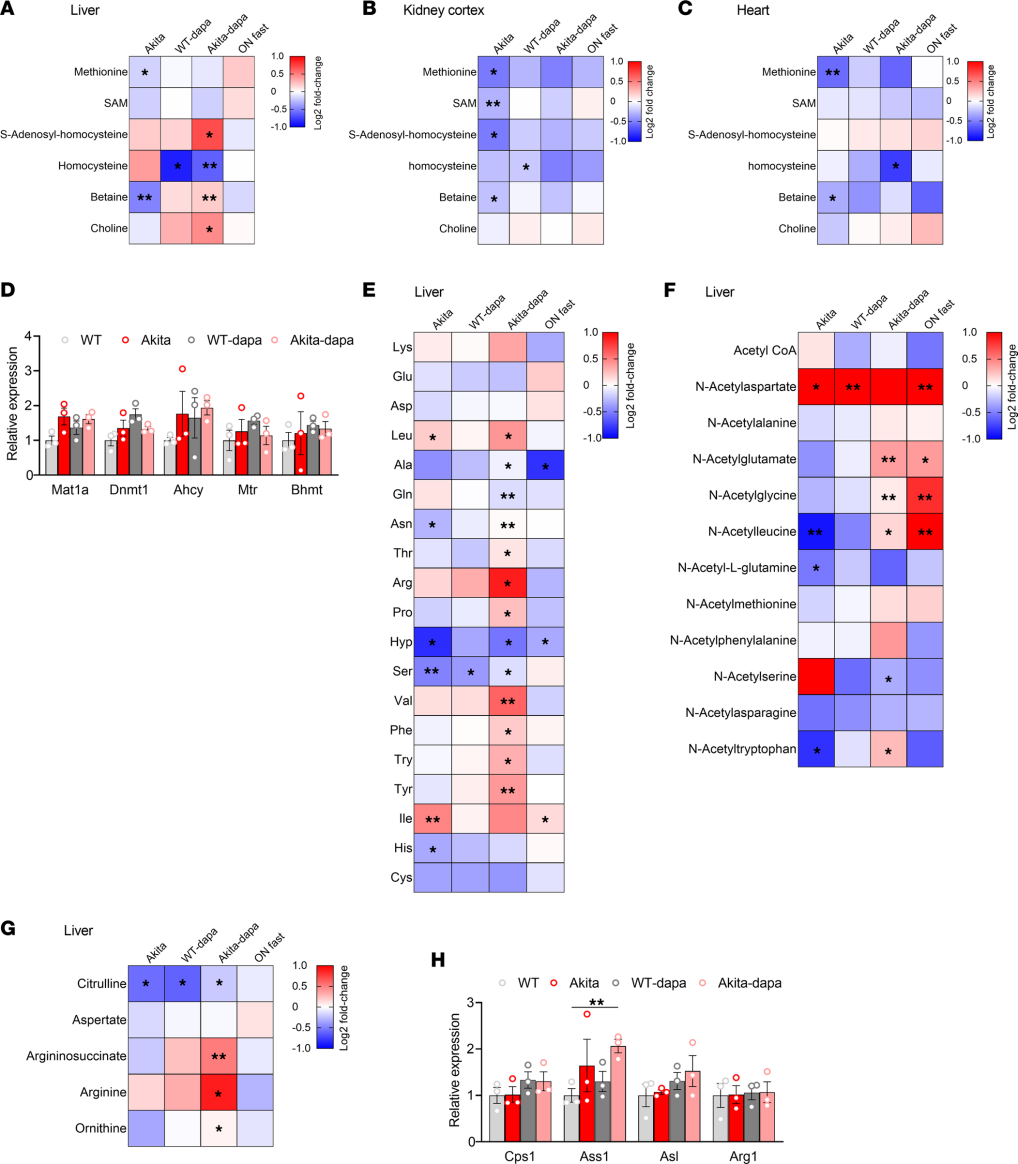
Effects of diabetes and of treatment with SGLT2i on one carbon and amino acid metabolism. (**A**–**C**) Heatmaps showing the relative levels of one carbon pathway metabolites in liver (**A**), kidney cortex (**B**), and heart (**C**). (**D**) mRNA levels of methionine cycle-related enzymes in the liver. (**E**–**G**) Heatmaps showing the relative levels of hepatic amino acids (**E**), acetylated amino acids (**F**), and urea cycle metabolites (**G**). Each square represents the average metabolite log_2_ fold change relative to WT control mice. (**H**) mRNA levels of urea cycle enzymes in liver extracts. Data represent the mean ± SEM, *n* = 3–6 mice per group. Data were analyzed by 2-way ANOVA. *P < 0.05, **P < 0.01.

One-carbon metabolism comprises a series of interlinking metabolic pathways that include the methionine and folate cycles that are central to cellular function, providing methyl groups for the synthesis of DNA, polyamines, amino acids, creatine, and phospholipids. Pathway enrichment analysis shows that, in the liver, one carbon metabolism is altered in diabetes and is affected by treatment of diabetic animals with dapagliflozin. In the diabetic liver, homocysteine was increased, whereas betaine, which promotes remethylating homocysteine to methionine, was decreased (Figure 7A). S-adenosylhomocysteine (SAH) was increased in diabetic animals treated with dapagliflozin, while the methyl donor S-adenosylmethionine (SAM) and methionine remained unchanged (Figure 7A). In addition, both methionine and betaine were decreased in the diabetic kidney and heart (Figure 7, B and C). Treatment with dapagliflozin increased hepatic betaine and decreased homocysteine in both normoglycemic and diabetic animals (Figure 7A). The expression of genes that regulate the methionine cycle were neither affected by diabetes nor by SGLT2i (Figure 7D); thus, the changes in metabolite levels may result from alterations in substrate availability or from effects on enzyme protein level, localization, and/or activity irrespective of gene expression. Of note, insulin similarly increased SAH, betaine, and choline as well as methionine, and it decreased homocysteine (Supplemental Figure 11A). Interestingly, elevated homocysteine has been reported to be involved in cardiovascular disease, steatohepatitis, and diabetic kidney disease (28–30), suggesting a potential mechanistic link between SGLT2i effects on hepatic one carbon metabolism and its protective effects.

### SGLT2i effects on amino acids and urea cycle.

In the kidney, there was a trend for decreased amino acid levels, except for the branched chain amino acid (BCAA) isoleucine and aspartate that were increased; there were no significant effects of dapagliflozin (not shown). In the diabetic liver, isoleucine was increased, whereas hydroxyproline and serine were downregulated (Figure 7E). Treatment of diabetic mice with dapagliflozin was associated with increased BCAAs (leucine, isoleucine, and valine), tyrosine, lysine, and arginine levels. In diabetes, amino acid acetylation was reduced and treatment with dapagliflozin increased the acetylation of several amino acids, including glutamate, leucine, phenylalanine, and tryptophan (Figure 7F). Increased amino acid metabolism and degradation requires ammonia disposal via the urea cycle, which converts toxic ammonia to urea. Consistently, we found the levels of urea cycle intermediates, including citrulline, arginosuccinate, arginine, and ornithine, increased in diabetic animals treated with dapagliflozin, compared with untreated Akita mice (Figure 7G). This was associated with increased expression of *Arginosuccinate synthetase* (*Ass1*), whereas the gene expression of other urea cycle enzymes was unchanged (Figure 7H).

Conversely, insulin markedly increased hepatic amino acid levels as well as glucose-derived amino acid synthesis, although the effects on amino acid acetylation and urea cycle metabolites were not consistently different compared with those induced by SGLT2i (Supplemental Figure 11, B–E).

### SGLT2i modulate AMPK-mTORC1 signaling in the kidney, liver, and heart.

**Figure 8:**
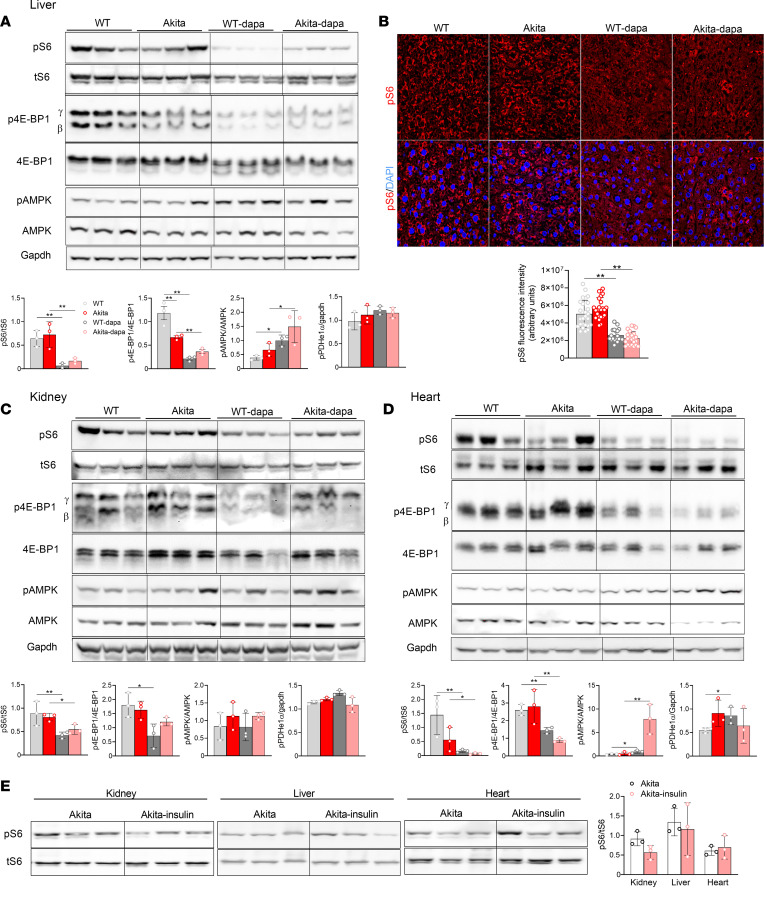
SGLT2i effects on mTORC1 and AMPK activity in WT and Akita mice. (**A**) Western blotting on liver extracts for phosphorylated S6 (pS6), tS6, phosphorylated 4E-PB1, 4E-BP1, phosphorylated AMP-activated protein kinase (pAMPK), AMPK, and GAPDH. (**B**) Immunofluorescence staining for pS6 on liver sections of 2-month-old WT and Akita mice treated with and without dapagliflozin. Total original magnification, 60***×***. (**C**) Western blotting on kidney extracts for pS6, tS6, phosphorylated 4E-PB1, 4E-BP1, phosphorylated AMPK (pAMPK), AMPK, and GAPDH. (**D**) Western blotting on heart extracts for pS6, S6, phosphorylated 4E-PB1, 4E-BP1, pAMPK, AMPK, and GAPDH. (**E**) Western blotting for pS6 and tS6 on kidney, liver, and heart extracts of Akita mice treated with and without insulin. Representative blots and quantifications are shown. Data represent the mean ± SEM, *n* = 3–6 mice per group. Data were analyzed by 2-way ANOVA (**A**–**D**) or by unpaired Student’s *t* test (**E**). *P < 0.05, **P < 0.01.

We and others have previously reported that SGLT2i inhibit mTORC1 activity in diabetic kidneys, thereby preventing kidney dysfunction and fibrosis (23, 24). Glycolytic metabolites that were altered by SGLT2i — e.g., DHAP and GA3P — regulate mTORC1 (31). Considering this and the findings that SGLT2i have robust effects on nutrients metabolism, we tested the effects of dapagliflozin treatment on AMPK-mTORC1 signaling in the kidney, liver, and heart of normoglycemic and diabetic animals. Dapagliflozin robustly decreased mTORC1 activity, evident by S6 and 4EBP1 phosphorylation, in all organs both in normoglycemic and in diabetic animals (Figure 8, A–D). Despite the fact that insulin acutely stimulates mTORC1, chronic treatment with basal insulin for 5 days did not affect steady-state S6 phosphorylation in the different organs (Figure 8E), and this contrasts the marked inhibition induced by SGLT2i. AMPK phosphorylation was increased in the liver and heart extracts of mice treated with dapagliflozin (Figure 8, A, C, and D). Overnight fasting partially mimicked the effects of SGLT2i on AMPK-mTORC1 signaling; AMPK phosphorylation was increased in the kidney cortex, liver, and heart extracts, whereas mTORC1 was inhibited in the heart only (Supplemental Figure 12).

## Discussion

We tested the hypothesis that reducing systemic glucose load by inducing glycosuria using a highly selective SGLT2i alters nutrient metabolism in the kidney, where it acts; the liver, which is an important nutrient sensor regulating whole-body glucose homeostasis; and the heart. We show here that treatment with SGLT2i induces metabolic reprogramming, orchestrated by AMPK-mTORC1 signaling, along with alterations in glucose, amino acid, and fat metabolism in the kidney, liver, and heart. Part of the metabolic effects were tissue dependent and were affected by the metabolic state — i.e., hyperglycemia and insulin deficiency.

AMPK-mTORC1 signaling generates a switch between anabolism and catabolism. Activation of AMPK along with inhibition of mTORC1 has been shown to promote energy production via lipolysis and fatty acid oxidation and via increased protein turnover through autophagy (32–34). We found that treatment with SGLT2i activated AMPK in the liver and heart, more so in diabetic animals, and induced marked inhibition of mTORC1 in both normoglycemic and diabetic animals. Consistently, previous studies showed that SGLT2i stimulate lipolysis and ketogenesis, which was observed mainly in the postabsorptive state (2, 14). Herein, we obtained a snapshot of nutrient metabolism in the fed state in animals that were treated with SGLT2i compared with controls. Under these conditions, the effects on lipid metabolism were relatively small; however, there were marked effects on carbohydrate and amino acid metabolism.

### Altered glucose metabolism in diabetes and effects of SGLT2i.

^13^C-glucose labeling showed that, in diabetes, glycolysis is dysregulated and there is impaired glucose oxidation. Remarkably, the downstream glycolytic intermediates pyruvate and lactate were decreased in the kidney, liver, heart, and plasma, suggesting that glycolysis was inhibited despite increased expression of several glycolytic enzymes. This finding stresses that enzyme gene expression does not necessarily reflect glycolytic flux. In the liver, the switch in the expression of glucose-sensing enzymes from glucokinase to hexokinase 2 may contribute to inhibition of glycolysis as hexokinase 2, whereas glucokinase is not inhibited by its product G6P; this may limit the availability of glycolytic intermediates despite hyperglycemia. Glucose oxidation was impaired in all tissues, as evident by decreased levels of ^13^C-labeled TCA cycle intermediates. Of note, acetyl CoA levels were consistently increased in all organs and ATP was preserved. In the heart, the levels of unlabeled TCA cycle intermediates were higher than in WT mice, suggesting a metabolic switch from glucose oxidation to alternative fuels, such as FFAs and ketone bodies that can feed the TCA cycle. This scenario is supported by the finding that the expression of the fatty acid transporter CD36 was increased in the diabetic liver.

Treatment with SGLT2i did not affect glucose-derived pyruvate and lactate levels, indicating that it failed to rescue glycolysis. Furthermore, treatment with dapagliflozin decreased pyruvate kinase activity in the liver of both normoglycemic and diabetic animals, suggesting greater inhibition of hepatic glycolysis by SGLT2i. Importantly, treatment with SGLT2i uniformly enhanced glucose oxidation in the various tissues, and it decreased expression of hepatic CD36, suggesting that, in diabetes, SGLT2i shift TCA cycle metabolism from fatty acid to glucose oxidation. These findings are consistent with a previous report showing that treatment with the SGLT2i empagliflozin increased glucose oxidation in the isolated working heart (35).

### SGLT2i effects on amino acid metabolism.

Treatment with SGLT2i was associated with increased levels of hepatic amino acids, as well as acetylated amino acids and urea cycle metabolites. This may reflect increased protein turnover and amino acid degradation, which is expected under conditions where mTORC1 is inhibited, leading to increased protein degradation via autophagy and the proteasome (34). Treatment with SGLT2i increased the levels of the ketogenic amino acids leucine, lysine, and tyrosine. In addition, the bulk of amino acids was unlabeled with ^13^C, indicating that they were not derived from glycolytic metabolites. On the contrary, insulin markedly increased the levels of glycogenic amino acids in all tissues.

Collectively, we suggest that SGLT2i increase the energetic efficiency and promote metabolic flexibility by increasing glucose and ketogenic amino acid oxidation in the postprandial state and fatty acid oxidation in the postabsorptive state. On the contrary, insulin increased glycolysis, inhibited ketogenesis, and enhanced glucose-derived amino acid synthesis and potentially lipogenesis, which are energy consuming.

It is commonly believed that, in diabetes, increased glucose oxidation leads to excess generation of reactive oxygen species and oxidative stress, consequently inducing tissue damage and vascular complications. The finding that SGLT2i increase glucose oxidation in multiple tissues while preventing tissue damage calls into question this paradigm. Increased glucose oxidation may, in fact, promote defense mechanisms through generation of NADPH via anaplerosis/cataplerosis shuttles. Indeed, we found that, in the kidney, treatment with SGLT2i was associated with increased NADPH/NADP and NADH/NAD ratios. Consistently, it has been previously shown that increased glycolysis along with oxidative phosphorylation was associated with prevention of diabetic kidney disease (26). Similarly, in the heart, enhanced glucose metabolism through cardiac specific activation of *Hexokinase 2* attenuated, rather than enhanced, cardiac hypertrophy by increasing pentose pathway flux, thereby increasing NADPH (36).

### SGLT2i effects on one carbon metabolism and the methionine cycle.

The one carbon metabolism is vital for cellular homeostasis due to its key functions in methionine and folate metabolism, protein, DNA, and RNA synthesis and its functions in redox state through effects on methionine and homocysteine balance to prevent cellular dysfunction and oxidative stress (37, 38). Polymorphisms in the *MTHFR* gene have been associated with various diseases, including diabetes, vascular disorders, and cancer (38). Betaine (N, N, N-trimethylglycine) is an amino acid derivative exerting numerous beneficial effects on the organism through alternative recycling of homocysteine to methionine. Interestingly, we found that betaine and methionine are reduced in the kidney, liver, and heart of young diabetic animals prior to development of end-organ complications. These findings are consistent with previous reports showing that the tissue content of betaine is reduced in type 2 diabetes (39). Furthermore, it has been recently reported that, in the SURDIAGENE (SURvival DIAbetes and GENEtics) study with a large, prospective cohort of patients with type 2 diabetes, betaine and homocysteine were associated with hospitalization for heart failure and/or mortality and that homocysteine was an independent predictor of all-cause mortality (30). Rodent studies show that betaine supplementation ameliorated oxidative and ER stress and reduced inflammation, in part, through activation of AMPK (39). Of note, we found that treatment with SGLT2i as well as insulin increased hepatic betaine and decreased homocysteine levels. Further studies are required to test the hypothesis that dysregulated betaine metabolism is involved in the pathophysiology of kidney, liver, and heart disease and that the beneficial effects of SGLT2i are partially mediated via modulation of hepatic betaine metabolism.

### Implications of mTORC1 inhibition for diabetes complications and aging.

Multiple studies in the past 2 decades show that dysregulated mTORC1 plays a critical role in the pathophysiology of kidney diseases, NAFLD, cardiovascular and neuronal disorders, and cancer (23–25, 40). Fine-tuned, moderate inhibition of mTORC1 is beneficial in that it reduces the cellular workload and stimulates autophagy and mitophagy, which improve protein and organelle quality control, thereby reducing cellular stress and inflammation. We previously reported that partial inhibition of mTORC1 in KPTCs by conditional heterozygous KO of *Raptor* was sufficient to prevent kidney fibrosis and dysfunction in diabetes (23). Selective inhibition of mTORC1 through deletion of the RagC/D guanosine triphosphatase–activating protein folliculin arm of mTORC1 has been recently shown to inhibit de novo lipogenesis and protect mice from NAFLD (40). Furthermore, mTORC1 inhibition by calorie restriction and treatment with low-dose rapamycin have been shown to extend lifespan and reduce age-related pathologies, including cardiomyopathy and cancer (41, 42). Notably, mTORC1 activity was not increased in kidney, liver, and heart extracts of young diabetic mice. We have previously shown that, in diabetes, mTORC1 is specifically activated in KPTCs and that the development of DKD in this model is strictly dependent on mTORC1 activation in these cells (23). Similarly, in pancreatic islets, stimulation of mTORC1 by hyperglycemia has been recently shown to impair glucose oxidation and β cell function (43). Collectively, these findings may suggest that hyperglycemia stimulates mTORC1 in a time- and cell type–dependent manner. It has been previously shown that age-associated alterations in mTORC1 activity is not uniformly increased in all tissues (44). Nevertheless, genetic and pharmacological inhibition of mTORC1 extend lifespan and delay the onset of age-related pathologies, irrespective of mTORC1 activation (41, 42). Therefore, SGLT2i may prevent the kidney and cardiovascular complications of diabetes and may promote healthy aging even in the absence of ubiquitous activation of mTORC1.

### Study limitations.

First, we used a single tracer and tested the metabolic effects in the fed state only. However, previous studies already show that treatment with SGLT2i promotes lipolysis and ketogenesis while fasting; we therefore mainly focused on the metabolic effects of SGLT2i on glucose metabolism in the fed state.

Second, we did not perform metabolomics and metabolic flux analyzes in other important tissues, such as adipose tissue, muscle, islets, and the brain.

Third, the kidney, liver and heart are complex organs that include multiple cell types. Therefore, the findings reflect the changes in the whole organ, and heterogeneity in the metabolic response in different tissues within the same organ can be overlooked. The current approach does not address intraorgan changes in metabolism — e.g., possible differences in the metabolic response in proximal and distal tubular cells. Nevertheless, our system’s biology approach enabled us to identify robust metabolic alterations with implications for the pathophysiology and treatment of diabetes.

In summary, we show here that diabetes leads to inhibition of glycolysis and glucose oxidation that can be accompanied by increased fatty acid transport and utilization for energy production. SGLT2i modulate AMPK-mTORC1 signaling in various organs, thereby facilitating glucose oxidation. We suggest that crosstalk between hexose and one carbon metabolism and AMPK-mTORC1 signaling mediate organ damage in diabetes and mediate the protective effects of SGLT2i in patients with and without diabetes. The finding that treatment with SGLT2i induces multiorgan inhibition of mTORC1 has far-reaching implications, not only for the treatment of kidney diseases, NAFLD, and heart failure, but also potentially for aging and cancer. Further preclinical and clinical studies assessing the effects of SGLT2i on aging and lifespan are warranted to assess whether this simple and safe therapy can be used to promote healthy aging.

## Methods

### Animals.

Mice were housed in the Hebrew University animal care unit with 12-hour light/dark cycles and fed standard chow diet and water ad libitum. Eight-week-old male WT C57BL/6 mice fed regular chow and diabetic male Akita (Ins2WT/C96Y) mice (The Jackson Laboratory) were treated with dapagliflozin (10 mg/kg/day in drinking water; Forxiga, AstraZeneca) and compared with untreated controls for 1–2 weeks. When specified, Akita mice were given a s.c. injection of insulin (2–4 U/day, degludec, Novo Nordisk) for 5 days. Mice were sacrificed by cervical dislocation, and the kidneys, liver, heart, and skeletal muscle were removed quickly, snap frozen in liquid nitrogen, and stored at −80°C prior to use.

### Continuous blood glucose monitoring.

Continuous blood glucose monitoring was performed using a FreeStyle Libre flash monitoring device (Abbott Diabetes Care) during the dark and light periods prior to and following treatment with dapagliflozin. The device, which is commonly used for continuous blood glucose monitoring in patients with diabetes, is composed of a sensor and a data reader. The sensor cannula traverses the skin with its tip in the interstitial fluid; the data are collected and saved by the reader. The mice were anesthetized by i.p. injection of ketamine (100 mg/kg body weight)/xylazine (10 mg/kg body weight) solution (both from Sigma-Aldrich). A small longitudinal incision was made in the middle of the low back after shaving and local disinfection; the sensor cannula was inserted, and the surface adhesion surface was sutured to the skin. Mice carrying the continuous glucose monitoring system (CGM) device were housed individually to prevent dislodging of the device. Readings were obtained starting 48 hours after recovery from surgery during 24 hours and then for additional 72 hours after adding dapagliflozin to the drinking water. Continuous glucose monitor plots displaying glucose measurements obtained in 15-minute intervals were generated using the Libre 1 Freestyle software.

### Metabolic tests.

Metabolic tests were performed in WT and Akita mice fed on regular chow that were treated with or without dapagliflozin. Dynamic tests were performed after 4-hour, 6-hour, or overnight fast as described in the figure legends. IPGTT was performed by i.p. injection of glucose (1.5 g/kg) followed by consecutive measurements of tail blood glucose at the indicated time points. ITT was performed by i.p. injection of insulin (1 U/kg). Hepatic glucose production was measured by i.p. injection of pyruvate (2 g/kg), glycerol (2 g/kg) (both from Sigma-Aldrich), and glucagon (100 mg/kg; Sigma-Aldrich). Blood glucose was measured by tail bleeds using Accu-Chek glucose meter (Roche Diagnostics GmbH). For measurement of serum insulin and glucagon, blood samples were collected from the facial vein in the fed state and after 4-hour fast. Insulin and glucagon were analyzed using ultrasensitive ELISA kits (Mercodia and Crystal Chem Inc.). Serum FGF21 levels after a 4-hour fast were measured by ELISA kit (R&D Systems, MF2100).

### LC-MS metabolomics analysis.

For measurements of kidney, liver, and heart metabolites, mice were fasted for 1 hour and then received 3 consecutive i.p. injections of ^13^C_6_-D-glucose (1 mg/g) (Sigma-Aldrich) at time points 0, 20, and 30 minutes. Mice were sacrificed 10 minutes after the last injection by cervical dislocation. A control group of mice was fasted overnight for 16 hours. Tissues were collected rapidly, snap frozen in liquid nitrogen, and stored at −80°C. For analysis of plasma metabolites, about 30 μL of blood was drawn from the tail into heparin-coated tubes immediately before the sacrifice of the mice. Plasma was separated and snap frozen in liquid nitrogen and kept at –80°C.

Frozen kidney cortex, livers, and hearts weighing ~30 mg were transferred into soft tissue homogenizing CK 14 tubes containing 1.4 mm ceramic beads (Bertin Corp.) prefilled with 300 μL of cold (–20°C) metabolite extraction solvent (methanol/acetonitrile/H_2_O, 50:30:20) and kept on ice. Samples were homogenized using Precellys 24 tissue homogenizer (3 cycles of 20 seconds at 6,000 rpm, with a 30-second gap between each of cycles; Bertin Technologies) cooled to 4°C. Homogenized extracts were centrifuged in the Precellys tubes at 18,000*g* for 15 minutes at 4°C; supernatants were collected in microcentrifuge tubes and centrifuged again at 18,000*g* for 10 minutes at 4°C. The supernatants were transferred to glass high performance liquid chromatography (HPLC) vials and kept at –75°C prior to LC-MS analysis. Plasma was diluted in a ratio of 1:10 with cold (–20°C) metabolite extraction solvent (methanol/acetonitrile, 75:25) and vortexed for 10 minutes. Samples were then treated as described above for tissue homogenized extracts and kept at –75°C prior to LC-MS analysis.

LC-MS metabolomics analysis was performed as described previously (45). Briefly, the Thermo Vanquish Flex ultra–high-performance liquid chromatography (UPLC) system coupled to Orbitrap Exploris 240 Mass Spectrometer (Thermo Fisher Scientific) was used. Resolution was set to 120,000 at 200 *m/z* with electrospray ionization and polarity switching mode to enable both positive and negative ions across a mass range of 67–1,000 *m/z*. The chromatography was performed as described previously (45). Briefly, UPLC setup consisted of ZIC-pHILIC column (Merck). Biological extracts (5 μL) were injected, and the compounds were separated using a mobile phase gradient of 15 minutes, starting at 20% aqueous (20 mmol/L ammonium carbonate adjusted to pH 9.2 with 0.1% of 25% ammonium hydroxide)/80% organic (acetonitrile) and terminated with 20% acetonitrile. Flow rate and column temperature were maintained at 0.2 mL/min and 45°C, respectively, for a total run time of 27 minutes. All metabolites were detected using mass accuracy below 1 ppm. Thermo Xcalibur 4.4 was used for data acquisition. TraceFinder 4.1 was used for data analysis. Peak areas of metabolites were determined by using the exact mass of the singly charged ions. The peak areas of different metabolites were determined using Thermo TraceFinderTM 4.1 software, where metabolites were identified by the exact mass of the singly charged ion and by known retention time, using an in-house MS library built by running commercial standards of all detected metabolites. For data normalization, raw data files were processed with Compound Discoverer 3.1 to obtain total measurable ion peak intensities for each sample. Each identified metabolite intensity was normalized to the total intensity of the sample. Metabolite-Auto Plotter 2 (46) was used for data visualization during data processing. Heatmaps of significant metabolites and metabolite set enrichment analysis were generated using MetaboAnalyst 5.0 (https://www.metaboanalyst.ca/) (47).

### Western blotting.

Tissue homogenates were prepared in RIPA buffer (25 mmol/L Tris-HCl [pH 7.6], 150 mmol/L NaCl, 1% NP-40, 1% sodium deoxycholate, 0.1% SDS), and protein concentration was determined with Bradford protein assay (Bio-Rad). Samples were resolved by 10% or 8% SDS-PAGE and transferred to nitrocellulose membranes. After blocking, blots were incubated overnight with rabbit anti-pS6 (5364S, Cell Signaling Technology), tS6 (2217S, Cell Signaling Technology), pACC (3661S, Cell Signaling Technology), Akt (9272s, Cell Signaling Technology), pAKT (ser473; 4060, Cell Signaling Technology), 4E-BP1 (9644, Cell Signaling Technology), p4E-BP1 (9459, Cell Signaling Technology), pPDHe1α (Ser293; 31866, Cell Signaling Technology), pAMPK (Thr172; 2535, Cell Signaling Technology), AMPK (2532, Cell Signaling Technology), mouse anti-GAPDH (ab8245, Abcam), and α-tubulin (ab7291, Abcam) antibodies at 4°C. Anti–mouse– or anti–rabbit HRP–conjugated secondary antibodies (Jackson ImmunoResearch) were used for 1 hour at room temperature, followed by chemiluminescence detection using Clarity Western ECL Blotting Substrate (Bio-Rad). Relative band intensities were quantified by densitometric analysis using the ImageJ software (NIH).

### RNA extraction and quantitative PCR (qPCR).

Total RNA was extracted from kidney cortex, liver, and heart using TRI Reagent (Bio-Lab) according to manufacturer’s specifications. RNA purity and concentration were assessed using a Thermo Fisher Scientific 2000 spectrophotometer. cDNA was synthesized using 2 μg of RNA by reverse transcription (Applied Biosystems). The qPCR was performed with FastStart SYBR-Green Master (Applied Biosystems) using a QuantStudio 5 Real-Time PCR System (Applied Biosystems). Gene expression was normalized to *Actb* expression. qPCR primers used in this study were as follow: *Gck* forward (F): 5′-CATCAGGAGGCCAGTGTAAAG-3′, *Gck* reverse (R): 5′-TCCCAGGTCTAAGGAGAGAAAG-3′; *HK2* F: 5′-GCTGGAGGTTAAGAGAAGGATG-3′, *HK2* R: 5′-TGGAGTGGCACACACATAAG-3′; *Aldob* F: 5′-CCAGCCTTGCTATCCAAGAA-3′, *Aldob* R: 5′-GCACCTCTGGCTCAACAATA-3′; *Eno1* F: 5′-CCTAGAACTCCGAGACAATGATAAG-3′, *Eno1* R: 5′-AGAGCAGGCGCAATAGTTT-3′; *Pkm2* F: 5′-TCCGGACTGGACTCATCAA-3′, *Pkm2* R: 5′-GGATGTTCTCGTCACACTTCTC-3′; *Gapdh* F: 5′-CCCTTGAGCTAGGACTGGATAA-3′, *Gapdh* R: 5′-GGGCTGCAGTCCGTATTTATAG-3′; *Pdha1* F: 5′-ACTTGTGTGATGGTCAGGAAG-3′, *Pdha1* R: 5′-TGAAGCCATGTGCTCGATAG-3′; *Cs* F: 5′-CAGACCCTTACCTGTCCTTTG-3′, *Cs* R: 5′-CAGACAAGCACCTCCTGATT-3′; *Aco2* F: 5′-CAGGATCCACGAAACCAATCT-3′, *Aco2* R: 5′-GCTTGTCCACTGGATGAATCT-3′; *Idh2* F: 5′-GATGACATCTGTGCTGGTCTG-3′, *Idh2* R: 5′-CCTTCTGGTGTTCTCGGTAATG-3′; *Ogdh* F: 5′-TGGTGGAAGCACAACCTAAC-3′, *Ogdh* R: 5′-TACATGGTGCCCTCGTATCT-3′; *Fh1* F: 5′-GTTCGACACCTTTGGTGAATTG-3′, *Fh1* R: 5′-CCGTAGCACCTCCAATCTTAAA-3′; *Acadm* F: 5′-CAGCCAATGATGTGTGCTTAC-3′, *Acadm* R: 5′-CATACTCGTCACCCTTCTTCTC-3′; *Echs1* F: 5′-GGACTGTTACTCCAGCAAGTTC-3′, *Echs1* R: 5′-CCCACCAAGAGCATAACCATT-3′; *Hadh* F: 5′-CCAAGAAGGGAATTGAGGAGAG-3′, *Hadh* R: 5′-ACAAACTCATCTCCAGCCTTAG-3′; *Cpt1a* F: 5′-CCGTGAGGAACTCAAACCTATT-3′, *Cpt1a* R: 5′-CAGGGATGCGGGAAGTATTG-3′; *Cd36* F: 5′-CCTCTGACATTTGCAGGTCTATC-3′, *Cd36* R: 5′-GCATTGGCTGGAAGAACAAATC-3′; *Pdk1* F: 5′-CTACTCAACCAGCACTCCTTATT-3′, *Pdk1* R: 5′-CACGTCGCAGTTTGGATTTATG-3′; *Pdk2* F: 5′-TCAATCAACACACCCTCATCTT-3′, *Pdk2* R: 5′-ATAGGCGTCTTTCACCACATC-3′; *Pdk3* F: 5′-CTGGACTTCGGAAGGGATAATG-3′, *Pdk3* R: 5′-TTAACCTCTCTCATGGTGTTAGC-3′; *Pdk4* F: 5′-CATGAATCAGCACATCCTCATATTC-3′, *Pdk4* R: 5′-CTTGGACTACTGCTACCACATC-3′; *Mat1a* F: 5′-ACCTACGGGACCTCCAATAA-3′, *Mat1a* R: 5′-CAGATCCAAGTCCCTGACAATAA-3′; *Dnmt1* F: 5′-AGACCACTGTTCCTCCTTCTA-3′, *Dnmt1* R: 5′-CTCTACCTGGCTCACTACAAAC-3′; *Ahcy* F: 5′-AGCTGCAACATCTTCTCTACTC-3′, *Ahcy* R: 5′-ACCACAGGTACTCCTCATCT-3′; *Mtr* F: 5′-CCCTGTGCATTGACTCTTCTAA-3′, *Mtr* R: 5′-AGGCTGATGCTGTTGACTATG-3′; *Bhmt* F: 5′-TGATGAAGGAGACGCTTTGG-3′, *Bhmt* R: 5′-CCTCTAGCTGTTGGCGAAATA-3′; *Cps1* F: 5′-CTGAACTGTGGAGTGGAACTATT-3′, *Cps1* R: 5′-CCTGTCTTCTGTGGCCATAAT-3′; *Ass1* F: 5′-CACATCCCTGGAACTCTTCAT-3′, *Ass1* R: 5′-GTAGATACCTCGGGACTTCATTC-3′; *Asl* F: 5′-TGGACAAGGTTGCTGAAGAG-3′, *Asl* R: 5′-GCTTCACCGATGAGTTCCTT-3′; *Arg1* F: 5′-GATTATCGGAGCGCCTTTCT-3′, *Arg1* R: 5′-TGGTCTCTCACGTCATACTCT-3′; *Actb* F: 5′-GGCTGTATTCCCCTCCATCG-3′, *Actb* R: 5′-CCAGTTGGTAACAATGCCATGT-3′.

### Immunofluorescence staining.

Paraffin sections were deparaffinized and rehydrated, and antigen retrieval was performed using 10 mM citrate buffer (pH 6.0). The sections were stained with rabbit anti-pS6 (5364S, Cell Signaling Technology, 1:250) antibody followed by Cy3 Anti-rabbit secondary antibody (111-165-144, Jackson ImmunoResearch Laboratories). Cell nuclei were visualized with DAPI staining. Digital images were obtained with a Nikon A1R confocal microscope, and fluorescence intensity was quantified using the ImageJ software (NIH).

### Pyruvate kinase activity assay.

Liver and kidney cortex pyruvate kinase activity was measured using Pyruvate Kinase Assay Kit (Abcam, ab83432) according to the manufacturer’s protocol.

### Statistics.

Data are expressed as the mean ± SEM. Statistical analysis was performed using GraphPad Prism 8.0.2 (GraphPad Software Inc.). The differences between groups were analyzed by unpaired 2-tailed Student’s *t* test or 1-way or 2-way ANOVA followed by Bonferroni test. Differences were considered to be statistically significant at *P* < 0.05.

### Study approval.

This study was approved by the IACUC of the Hebrew University of Jerusalem (protocol no. MD-21-16585-4).

## Author contributions

GL and AKL conceived the study. GL, AKL, YR, IA, and EG designed experiments. AKL, YR, IA, LK, RBHS, DK, and LH performed experiments and analyzed results. OM, EC, DBZ, EBM, JT, and EG were involved in the analysis and interpretation of the data. BA performed experiments and analyzed results. GL, AKL, and YR wrote the paper.

## Supplementary Material

Supplemental data

Supplemental table 1

Supplemental table 2

Supplemental table 3

Supplemental table 4

## Version 1 02/21/2023

In-Press Preview

## Version 2 04/10/2023

Electronic publication
