# Supplementary material for: Mapping the metabolic reprogramming induced by sodium-glucose cotransporter 2 inhibition
Source: JCI Insight. 2023 Apr 10;8(7):e164296. doi: 10.1172/jci.insight.164296 (PMC10132155; doi:10.1172/jci.insight.164296)
Supplement: Supplemental data [file jciinsight-8-164296-s215.pdf]

Supplemental material

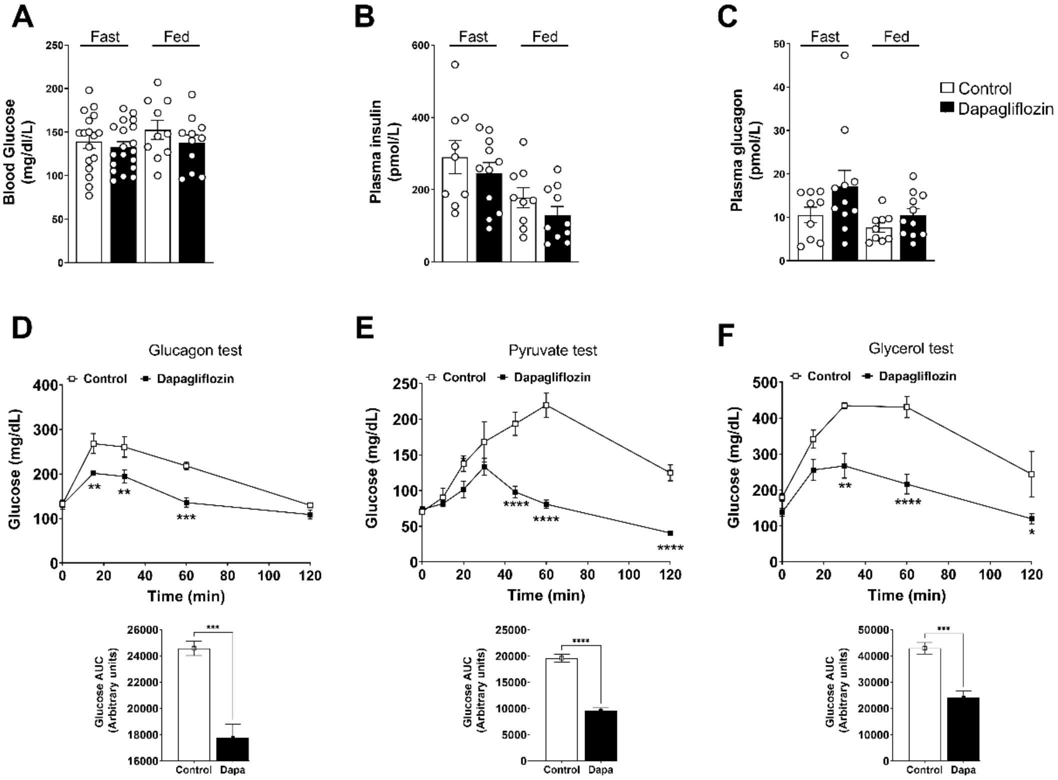

Figure S1. Effects of SGLT2i treatment on fed and fasting blood glucose, serum insulin and glucagon levels, and on hepatic glucose production. Two-month-old WT mice were fed on regular chow with or without dapagliflozin (10 mg/kg/day in drinking water) for 2 weeks. Plasma glucose **(A)**, insulin **(B)** and glucagon **(C)** levels in the fed state and following a 4 h fast are shown. Data represent the mean ± SEM of 4-11 mice per group. **(D)** Glucagon test; glucagon (100 µg/kg) was injected IP after 6 h fast. **(E)** Pyruvate tolerance test; pyruvate (2 g/kg) was injected IP after an overnight fast. **(F)** Glycerol tolerance test; glycerol (2 g/kg) was injected IP after 6 h fast. Data represent the mean ± SEM of 3-4 mice per group. *<0.05, **<0.005, ***<0.0005, ****< 0.0001.

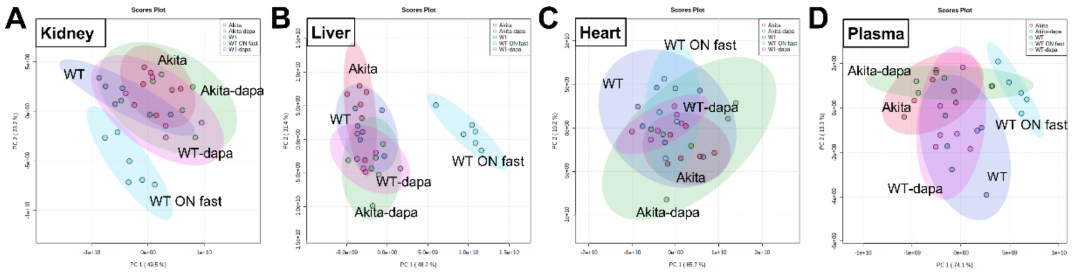

Figure S2. PCA score plots of metabolite abundance in WT and Akita mice treated with and without dapagliflozin for one week and in overnight-fasted WT mice. (**A**) kidney cortex, (**B**) liver (**C**) heart and (**D**) plasma**.** Each dot represents one biological replicate. n = 6 mice per group.

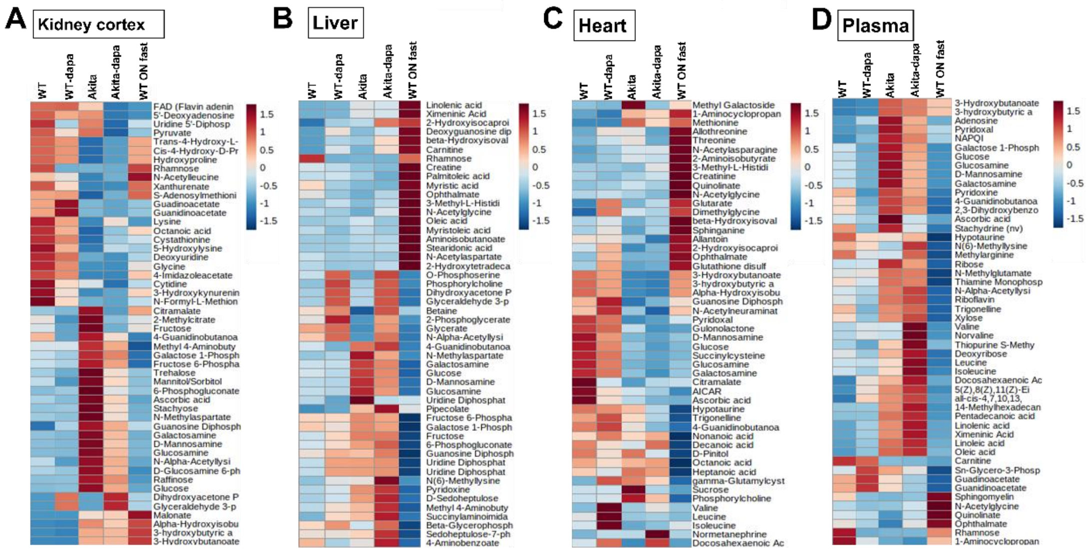

Figure S3. Effects of SGLT2i on metabolite levels in kidney, liver and heart of WT and Akita mice. WT and Akita mice were treated with or without dapagliflozin for one week. Overnight-fasted WT mice were used as an additional control. (**A-D**) Heatmaps showing the top 50 metabolites that were differentially expressed in diabetes and in response to treatment with dapagliflozin (dapa) or after an overnight fast: (**A**) Kidney cortex, (**B**) liver, (**C**) heart and (**D**) plasma. Each square represents the average metabolite abundance. n = 6 mice per group.

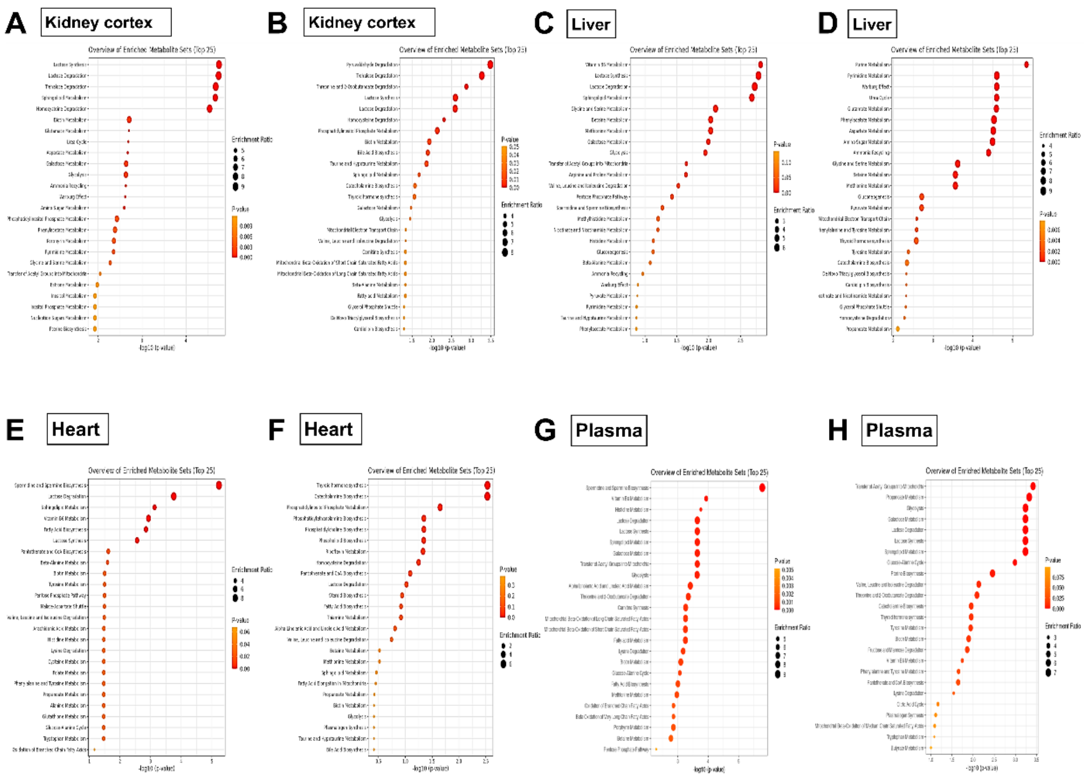

Figure S4. Metabolomics-based pathway enrichment analysis in kidney, liver, heart and plasma. (**A-B**) Pathway enrichment analysis in kidney extracts: (**A**) WT compared to Akita mice and (**B**) Akita compared to Akita mice treated with dapagliflozin. (**C-D**) Pathway enrichment analysis in liver extracts: (**C**) WT compared to Akita mice and (**D**) Akita compared to Akita mice treated with dapagliflozin. (**E-F**) Pathway enrichment analysis in heart extracts: (**E**) WT compared to Akita mice and (**F**) Akita compared to Akita mice treated with dapagliflozin. (**G-H**) Pathway enrichment analysis in plasma: (**G**) WT compared to Akita mice and (**H**) Akita compared to Akita mice treated with dapagliflozin. n = 6 mice per group.

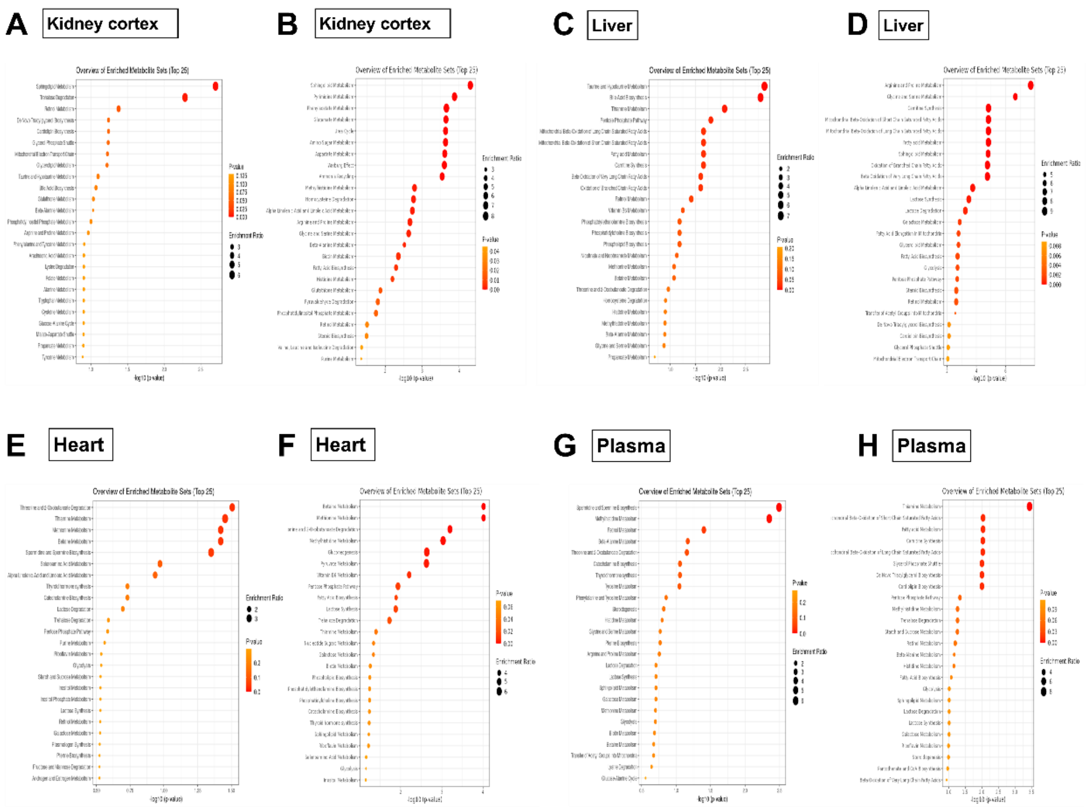

Figure S5. Metabolomics-based pathway enrichment analysis in kidney, liver, heart and plasma. (**A-B**) Pathway enrichment analysis in kidney extracts: (**A**) WT mice treated with dapagliflozin compared to controls and (**B**) WT mice fasted overnight compared to untreated control mice in the fed state. (**C-D**) Pathway enrichment analysis in liver extracts: (**C**) WT mice treated with dapagliflozin compared to untreated controls and (**D**) WT mice fasted overnight compared to control mice in the fed state. (**E-F**) Pathway enrichment analysis in heart extracts: (**E**) WT mice treated with dapagliflozin compared to untreated controls and (**F**) WT mice fasted overnight compared to control mice in the fed state. (**G-H**) Pathway enrichment analysis in plasma: (**G**) WT mice treated with dapagliflozin compared to untreated controls and (**H**) WT mice fasted overnight compared to control mice in the fed state. n = 6 mice per group.

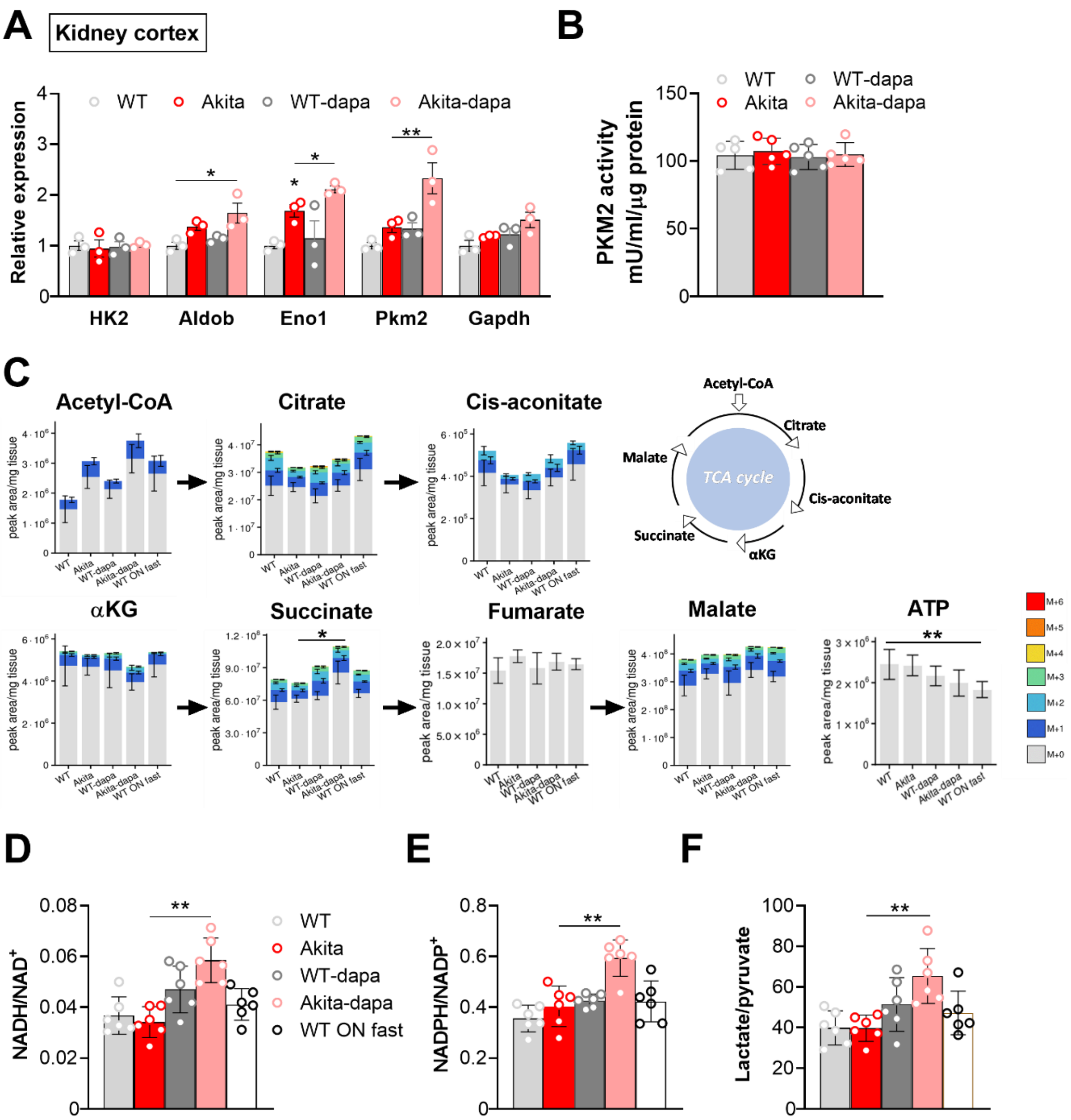

Figure S6. Effect of diabetes and of SGLT2i on glycolysis and glucose oxidation in kidney cortex of WT and Akita mice. WT and Akita mice were treated with and without dapagliflozin for one week followed by ^{13}C-glucose injections and metabolomics and metabolic flux analyses. (**A**) mRNA expression of glycolytic enzymes in kidney cortex. (**B**) Pyruvate kinase activity in kidney homogenates. (**C**) Levels of glycolytic intermediates in kidney cortex extracts. Shown are the relative levels of unlabeled (^{12}C) glucose and ^{13}C-labeled glycolytic intermediates. (**D-F**) NADH/NAD$^+$, NADPH/NADP$^+$, and lactate/pyruvate ratios in kidney cortex extracts. Data represent the mean ± SEM, n = 3-6 mice per group. For statistical analysis, we used the sum of the unlabeled + ^{13}C labeled metabolites. *$p < 0.05$, **$p < 0.01$.

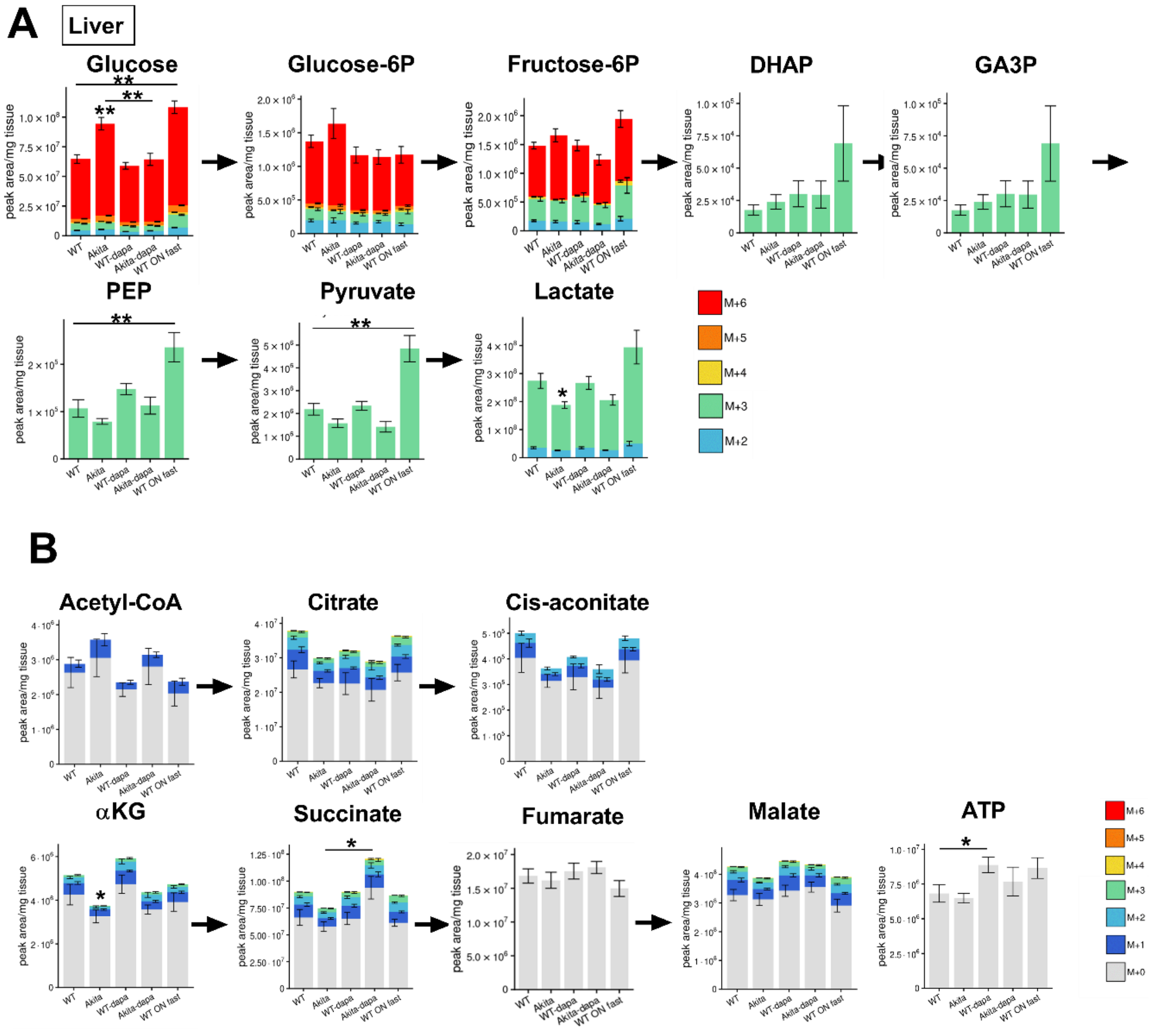

Figure S7. Effect of diabetes and of SGLT2i on glycolysis and glucose oxidation in liver of WT and Akita mice. WT and Akita mice were treated with and without dapagliflozin for one week followed by ^{13}C-glucose injections and metabolomics and metabolic flux analyses. (**A**) Relative abundance of ^{13}C-labeled glucose and glycolytic intermediates in liver. (**B**) Levels of glycolytic intermediates in kidney cortex extracts. Shown are the relative levels of unlabeled (^{12}C) glucose and ^{13}C-labeled glycolytic intermediates. Data represent the mean ± SEM, n = 4-6 mice per group. For statistical analysis, we used the sum of all ^{13}C isotopologues for each metabolite or the unlabeled + ^{13}C labeled metabolites. *$p < 0.05$, **$p < 0.01$.

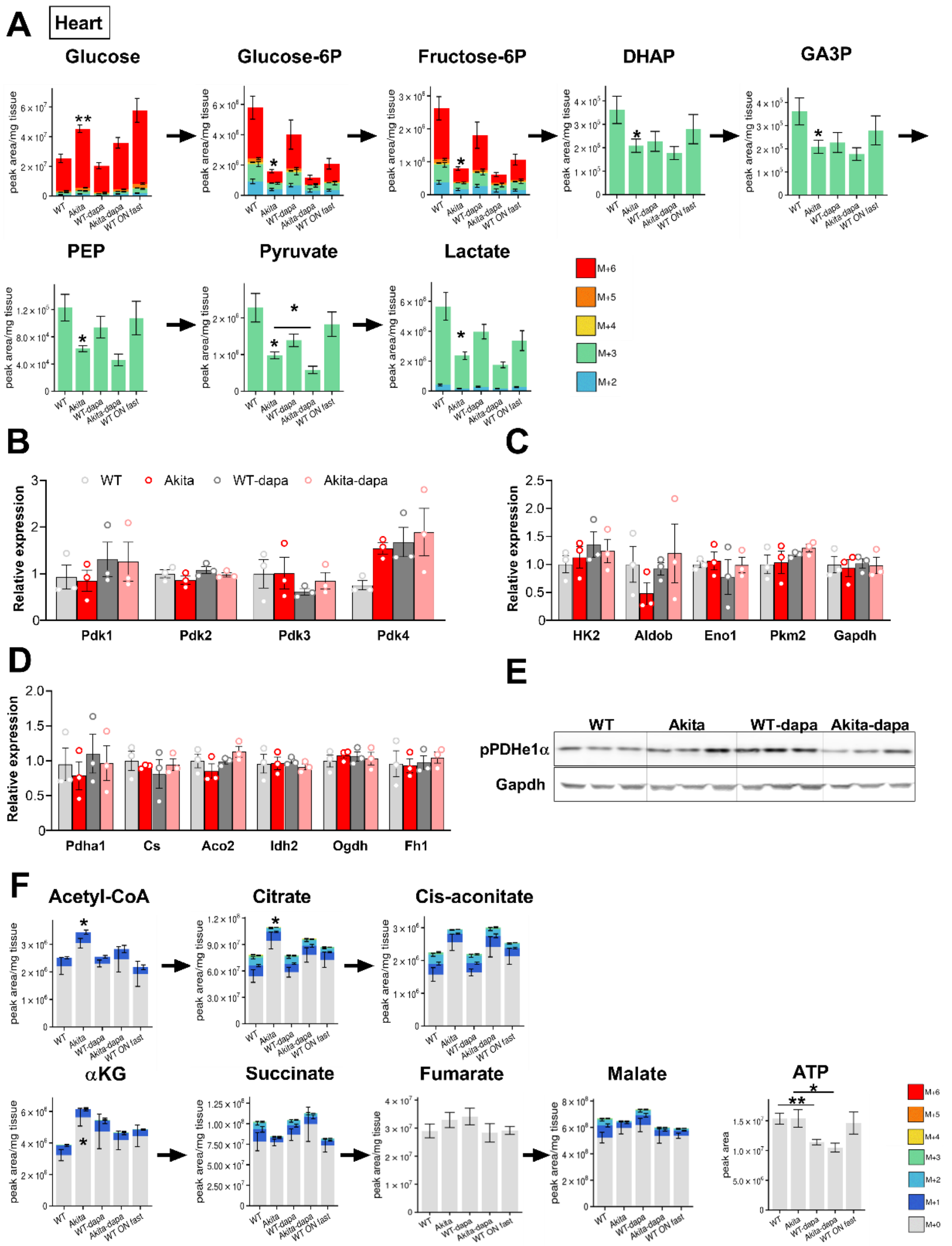

Figure S8. Effect of diabetes and of SGLT2i on glycolysis and glucose oxidation in heart of WT and Akita mice. WT and Akita mice were treated with and without dapagliflozin for one week followed by ^{13}C-glucose injections and metabolomics and metabolic flux analyses. (**A**) Relative abundance of ^{13}C-labeled glucose and glycolytic intermediates in heart. (**B**) mRNA levels of Pdk1-4 in heart. (**C**) mRNA expression of glycolytic enzymes in heart. (**D**) mRNA expression of TCA cycle enzymes in heart. (**E**) Western blotting on heart extracts for phosphorylated pyruvate dehydrogenase α1 (pPDHe1α) and GAPDH. (**F**) Levels of glycolytic intermediates in heart extracts. Shown are the relative levels of unlabeled (^{12}C) glucose and ^{13}C-labeled glycolytic

intermediates. Data represent the mean ± SEM, n = 3-6 mice per group. For statistical analysis, we used the sum of all ^{13}C isotopologues for each metabolite or the unlabeled + ^{13}C labeled metabolites. *$p < 0.05$, **$p < 0.01$.

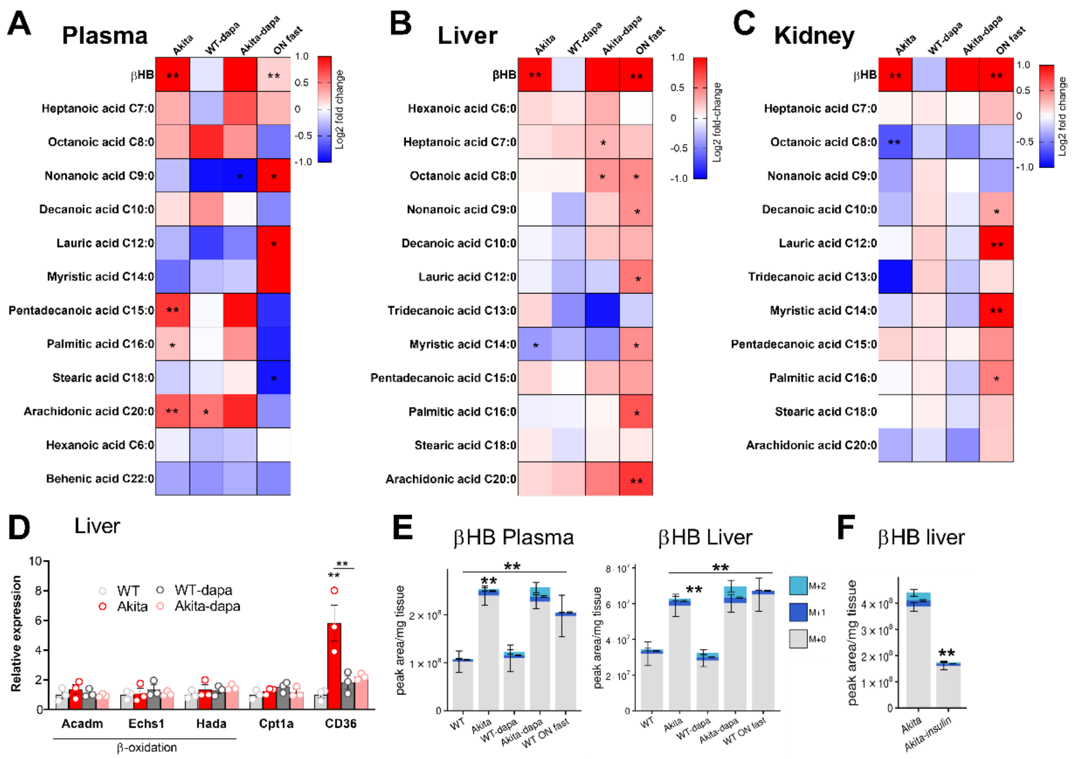

Figure S9. Effects of SGLT2i on fatty acid and ketone levels in WT and in Akita mice. (**A-C**) Heatmaps showing the relative levels of βHB and different free-fatty acids in (**A**) plasma, (**B**) liver and (**C**) kidney cortex. (**D**) mRNA levels of genes involved in β-oxidation and fatty acid transport and metabolism in the liver. (**E-F**) Levels of unlabeled (^{12}C) and ^{13}C-labeled βHB in liver and plasma extracts. (**F**) βHB levels in insulin-treated Akita mice compared to Akita controls. Data represent the mean ± SEM, n = 3-6 mice per group. For statistical analysis, we used the sum of unlabeled + ^{13}C-labeled metabolite levels. $^{*}p < 0.05$, $^{**}p < 0.01$.

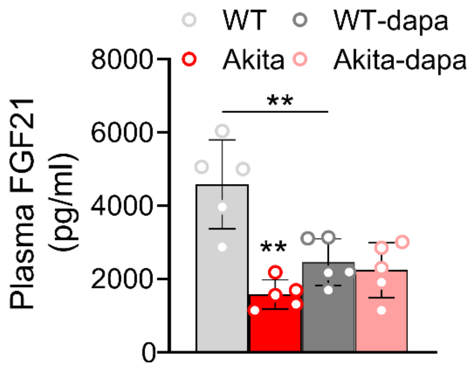

Figure S10. Plasma FGF21 levels in WT and Akita mice treated with and without dapagliflozin. Data represent the mean ± SEM, n = 5 mice per group. *$p < 0.05$, **$p < 0.01$.

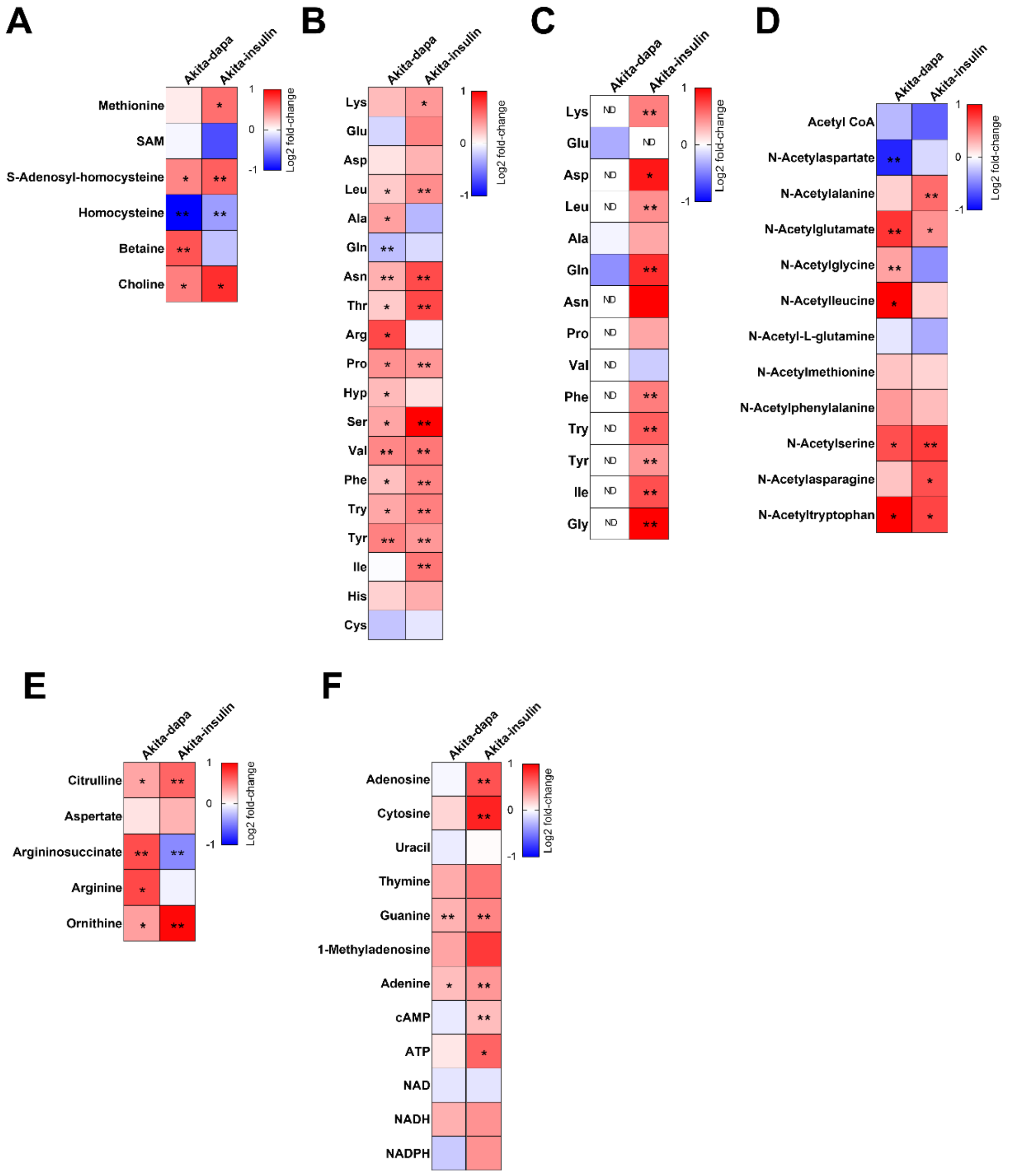

Figure S11. Effects of dapagliflozin and insulin on one-carbon and amino acids metabolism, acetylated amino acids, urea cycle, fatty acids and ketone levels in *Akita* mice. *Akita* mice were treated with and without insulin or dapagliflozin for 5 and 7 days, respectively, followed by ^{13}C-glucose injections and metabolomics and metabolic flux analyses. Heatmaps showing the relative levels of metabolites in liver extracts are shown. (**A**) one-carbon pathway metabolites, (**B**) amino acids, (**C**) ^{13}C-labeled amino acids, (**D**) acetylated amino acids, (**E**) urea cycle metabolites and (**F**) nucleic acids. Each square represents the average metabolite log2 fold change relative to untreated *Akita* mice. ND, not detected. Data represent the mean ± SEM, n = 6 mice per group. *$p < 0.05$, **$p < 0.01$.

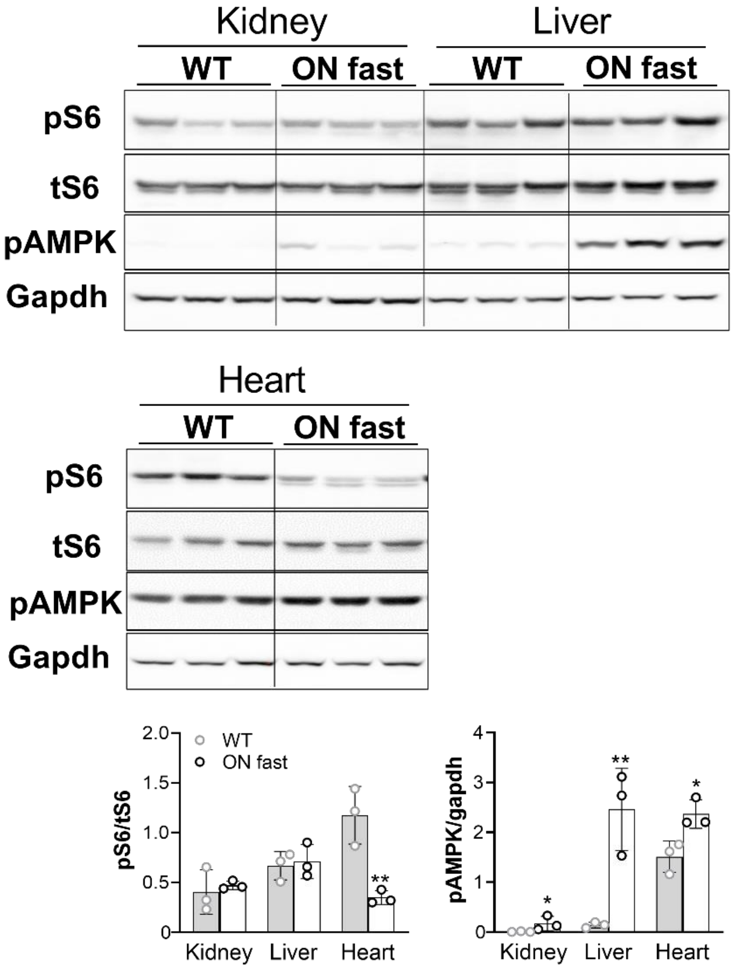

Figure S12. mTORC1 and AMPK activity in kidney, liver and heart in overnight fasted WT mice compared to fed control mice. Western blotting for pS6, S6, pAMPK and Gapdh on kidney, liver and heart extracts of WT mice after overnight fasting compared to controls. Representative blots and quantifications are shown. Data represent the mean ± SEM, n = 3 mice per group. *$p < 0.05$, **$p < 0.01$.
